# RNA design via structure-aware multifrontier ensemble optimization

**DOI:** 10.1093/bioinformatics/btad252

**Published:** 2023-06-30

**Authors:** Tianshuo Zhou, Ning Dai, Sizhen Li, Max Ward, David H Mathews, Liang Huang

**Affiliations:** School of Electrical Engineering and Computer Science, Oregon State University, Corvalli OR 97330, United States; School of Electrical Engineering and Computer Science, Oregon State University, Corvalli OR 97330, United States; School of Electrical Engineering and Computer Science, Oregon State University, Corvalli OR 97330, United States; Department of Computer Science and Software Engineering, The University of Western Australia, Perth, Australia; Department of Biochemistry and Biophysics, University of Rochester Medical Center, Rochester, NY 14642, United States; Center for RNA Biology, University of Rochester Medical Center, Rochester, NY 14642, United States; Department of Biostatistics & Computational Biology, University of Rochester Medical Center, Rochester, NY 14642, United States; School of Electrical Engineering and Computer Science, Oregon State University, Corvalli OR 97330, United States

## Abstract

**Motivation:**

RNA design is the search for a sequence or set of sequences that will fold to desired structure, also known as the inverse problem of RNA folding. However, the sequences designed by existing algorithms often suffer from low ensemble stability, which worsens for long sequence design. Additionally, for many methods only a small number of sequences satisfying the MFE criterion can be found by each run of design. These drawbacks limit their use cases.

**Results:**

We propose an innovative optimization paradigm, SAMFEO, which optimizes ensemble objectives (equilibrium probability or ensemble defect) by iterative search and yields a very large number of successfully designed RNA sequences as byproducts. We develop a search method which leverages structure level and ensemble level information at different stages of the optimization: initialization, sampling, mutation, and updating. Our work, while being less complicated than others, is the first algorithm that is able to design thousands of RNA sequences for the puzzles from the Eterna100 benchmark. In addition, our algorithm solves the most Eterna100 puzzles among all the general optimization based methods in our study. The only baseline solving more puzzles than our work is dependent on handcrafted heuristics designed for a specific folding model. Surprisingly, our approach shows superiority on designing long sequences for structures adapted from the database of 16S Ribosomal RNAs.

**Availability and implementation:**

Our source code and data used in this article is available at https://github.com/shanry/SAMFEO.

## 1 Introduction

Ribonucleic acid (RNA) plays essential roles in the core activities within living cells such as transcription and translation, catalyzing reactions, and controlling gene expression. Designing RNA molecules with specific functions or structures is an indispensable part of synthetic biology. One important and growing topic on synthetic RNA is noncoding RNA design (Hofacker et al. 1994; Taneda 2011; Churkin et al. 2018; Portela 2018; Shi et al. 2018), which also has profound applications in RNA-based therapeutics and diagnostics, including siRNA, antisense oligos, PCR primers, and CRISPR guide RNAs.

Given a target structure, RNA design aims to find sequences that can fold into that structure, in order to create artificial RNA molecules that have a desired function, such as artificial ribozymes (Bauer and Suess 2006), artificial miRNAs (Schwab et al. 2006), artificial RNA aptamers (Hamada 2018), and artificial riboswitches (Findeiß et al. 2017). This problem, however, has been proved NP-hard (Bonnet et al. 2020).

The simplest RNA design method is adaptive walk (Hofacker et al. 1994), which starts from a random sequence, uniformly chooses one or two positions to mutate at each step, and accepts it if and only if the objective becomes better than before. A variety of methods have been proposed to improve this process. To reduce the time cost from many evaluations for the whole sequence, RNAinverse (Hofacker et al. 1994; Lorenz et al. 2011) optimizes small substructures first and then proceeds to larger ones. Similarly, RNA-SSD (Andronescu et al. 2004) and NUPACK (Zadeh et al. 2011) adopt a hierarchical decomposition strategy. Recently, many methods apply genetic or evolutionary algorithm to RNA design, such as MODENA (Taneda 2011), Frnakenstein (Lyngsø et al. 2012), m2dRNAs (Rubio-Largo et al. 2019), and ERD (Esmaili-Taheri and Ganjtabesh 2015). Among them, MODENA and m2dRNAs use multi-objective function while Frnakenstein focuses on the multitarget inverse folding problem. MCTS-RNA (Yang et al. 2017) and NEMO (Portela 2018) use Monte Carlo search to reach a more extensive search space. DSS-Opt (Matthies et al. 2012), RNAiFold (Garcia-Martin et al. 2013), and antaRNA Kleinkauf et al. (2015) use various approaches to allow more constraints. Note that there are also some learning-based methods for RNA design (Eastman et al. 2018; Runge et al. 2018), which, however, are characterized by large amounts of training data and time, but have not shown superior effectiveness compared to optimization-based methods.

Despite various techniques that have been developed, there still exist some limitations in the current methods mentioned above. First, mainstream methods focus on the MFE criterion, i.e. finding sequences whose MFE structure is the same as the target structure. However, the sequence found by MFE criterion can often allow alternative structures with only slightly higher energies (Hofacker et al. 1994), causing a very low equilibrium probability for the sequence to fold into the target structure. Another related issue is that all the current methods ignore the case when a designed sequence has multiple MFE structures (Ward et al. 2022). In addition, most methods can only find one sequence or a small population of sequences within one run of design. However, it is more engaging to design as many as possible sequences in one time. Here we state that the benefits of finding a large amount of MFE solutions are 2-fold: (i) It is helpful to solve RNA design problem with more constraints, e.g. sequence compositions and GC content (Garcia-Martin et al. 2013; Esmaili-Taheri and Ganjtabesh 2015; Kleinkauf et al. 2015). (ii) It is crucial for further developing learning-based RNA design methods, for which one bottleneck is the lack of supervised training data especially the scarcity of MFE solutions.

To address the above drawbacks, we propose a new design paradigm *Structure-Aware Multifrontier Ensemble Optimization* (SAMFEO), which optimizes ensemble objectives to perform RNA Design. To handle the incompatibility between objective and MFE criterion, we generate the MFE solutions as byproducts of the iterative optimization process. In addition, we have invented a search method integrated with several components to utilize structure level and ensemble level information at different optimization stages to make the design more effective.

Our main contributions are as follows:

- We formulate RNA design as a general ensemble objective optimization problem, and propose to get MFE solutions from byproducts of optimization. As a result, not only more target structures can be designed successfully, but also for each target structure, a wide variety of MFE solutions can be found.
- We invent a structure-aware optimization process to make use of structure level and ensemble level information at different stages of optimization: initialization, sampling, mutation, and updating.
- Instead of performing greedy search to find suitable sequences, we adopt a multifrontier search framework to keep the running best *k* sequences in optimization process. This lead to even more satisfactory MFE solutions being found.
- Our work SAMFEO solves the most Eterna100 puzzles compared to other optimization based RNA design methods. The only RNA design method that can solve more puzzles than SAMFEO is dependent on heuristic rules coded by human experts. More importantly, the quality of MFE solutions designed by SAMFEO is higher than other methods. Experiments with 16S RNA structures demonstrate that SAMFEO exhibits an absolute advantage over baselines on long sequence design.

## 2 The RNA design problem

An RNA sequence *x* of length *n* is specified as a string of base nucleotides $x_{1}x_{2}\ldots x_{n},$ where $x_{i}\in \{A,C,G,U\}$ for i=1,2,…,n. A secondary structure P for *x* is a set of paired indices where each pair (i,j)∈P indicates two distinct bases $x_{i}x_{j}\in \{\text{CG},\text{GC},\text{AU},\text{UA},\text{GU},\text{UG}\}$ and each index from 1 to *n* can only be paired once. A secondary structure is pseudoknot-free if there don’t exist two pairs (i,j)∈P,(k,l)∈ P such that i<k<j<l. In short, a pseudoknot-free secondary structure is a properly nested set of pairings in an RNA sequence. Alternatively, P can be represented as a string $y=y_{1}y_{2}\ldots y_{n}$, where a pair of indices (i,j)∈ P corresponds to $y_{i}=“(”,\;y_{j}=“)”$ and any unpaired index *k* corresponds to $y_{k}=“)”$. The unpaired indices in *y* is denoted as unpaired(y) and the set of paired indices in *y* is denoted as pairs(y), which is equal to P. In nature, some RNA structures contain crossing pairings called pseudoknots. Since the computational model we use does not allow these, we do not consider them. Henceforth we elide pseudoknot-free secondary structure to just secondary structure or structure for brevity.

### 2.1 MFE and structure distance

The *ensemble* of an RNA sequence *x* is the set of all secondary structures that *x* can possibly fold into, denoted as Y(x). The *free energy* ΔG(x,y) is used to characterize the stability of y∈Y(x). The lower the free energy ΔG(x,y), the more stable the secondary structure *y* for *x*. The structure with the *minimum free energy* is the most stable structure in the ensemble, i.e. MFE structure,


(1) $$\text{MFE}(x)=\underset{y\in Y(x)}{\text{argmin}}\Delta G(x,y).$$

Notice that ties for the argmin are broken arbitrarily, thus there could be multiple MFE structures for given *x*. (Technically, MFE(*x*) should be a set.) The *partition function* sums the contribution of all structures defined as where *R* is the molar gas constant and *T* is the absolute temperature.


(2) $$Q(x)=\sum_{y\in Y(x)}e^{-\Delta G(x,y)/RT},$$

RNA design problem is actually the inverse problem of RNA folding. Given a target structure $y^{*}$, RNA design aims to find suitable RNA sequence *x* such that $\text{MFE}(x)=y^{*}$. However, the issue with multiple MFE structures is often overlooked in the literature. Almost all the published methods (Hofacker et al. 1994; Lorenz et al. 2011; Taneda 2011; Garcia-Martin et al. 2013; Rubio-Largo et al. 2019) take whatever arbitrary structure when there are multiple MFE structures. Here we follow a more strict definition of MFE criterion adopted in some previous studies (Haleš et al. 2015; Yao et al. 2019; Bonnet et al. 2020; Ward et al. 2022) on the designability of RNA to address this issue, i.e. *x* is a correct design if and only if y is the only MFE structure of *x*, which we call unique MFE(uMFE) criterion to differentiate it from the traditional MFE criterion. Formally, $\text{uMFE}(x)=y^{*}$ if and only if


(3) $$\Delta G(x,y^{*})<\Delta G(x,y),\;\forall y\in Y(x)\;\text{and}\;y\neq y^{*}.$$

From the perspective of optimization, the satisfaction of MFE criterion requires that the structure distance between target structure $y^{*}$ and MFE structure of *x* is minimized to 0. Therefore, many methods focus on optimizing $d(y^{*},\text{MFE}(x))$. The function d(y′,y″) represents the distance between two secondary structures y′ and y″, which is defined as


(4) d(y′,y″)=|x|−2·|pairs(y′)∩pairs(y″)|−|unpaired(y′)∩unpaired(y″)|.

### 2.2 Equilibrium probability

However, the objective of structure distance is not able to capture the equilibrium probability of a designed sequence folding into target structure, which is defined based on partition function,


(5) $$p(y|x)=\frac{e^{-\Delta G(x,y)/RT}}{Q(x)}.$$

In other words, the MFE structure of *x* is also the structure with the highest equilibrium probability in the ensemble. Nevertheless, the equilibrium probability $p(y^{*}|x)$ could be arbitrarily small even if $y^{*}$ is a MFE structure of *x*. Built upon the concept of equilibrium probability, the *base-pairing probability* of two positions *i* and *j* can be defined as the probability that positions *i*, *j* are paired in the ensemble,


(6) $$p_{ij}=\sum_{y\in Y(x),(i,j)\in \text{pairs}(y)}p(y|x).$$

### 2.3 Ensemble defect

Another approach to optimizing the probability of components of the designed structure is to minimize the *normalized ensemble defect*, the mean probability that a nucleotide is incorrectly structured in the folding ensemble (Dirks et al. 2004; Zadeh et al. 2011). The value of normalized ensemble defect is between 0 and 1 given by


(7) $$\text{NED}(x,y^{*})=\frac{1}{n}\;E_{y∼p(y|x)}\;d(y^{*},y)=\frac{1}{n}\sum_{y\in Y(x)}d(y^{*},y)\cdot p(y|x),$$

For brevity, ensemble defect is used as NED in the following sections. The naïve calculation of Equation (7) requires enumerating all possible structures in the ensemble, but by plugging Equation 4 we have where *q_j_* is the probability of *j* being unpaired, i.e. $q_{j}=1-\sum_{i}p_{ij}$. As a result, we can now use base-pairing probabilities to compute the ensemble defect. This also means that NED can be decomposed as the sum of each *positional defect*, denoted as $\epsilon_{i}(x,y^{*})$ for position *i*,


(8) $$\text{NED}(x,y^{*})=|x|-2\sum_{(i,j)\in \text{pairs}\left(y^{*}\right)}p_{ij}-\sum_{j\in \text{unpaired}\left(y^{*}\right)}q_{j},$$

(9) $$\epsilon_{i}(x,y^{*})=\{\begin{array}{cc} 1-q_{i}, & \text{if}\;i\in \text{unpaired}(y^{*});\\ 1-p_{ij}, & \text{if}\;(i,j)\in \text{pairs}(y^{*})\;\text{for some}\;j;\\ 1-p_{ji}, & \text{if}\;(j,i)\in \text{pairs}(y^{*})\;\text{for some}\;j. \end{array}$$

## 3 Structure-aware multifrontier ensemble optimization

First we formulate RNA design as a constrained optimization problem and introduce our basic optimization framework. Then we present four main stages by chronological order at each iterative step. Finally, we briefly discuss the complexity of our algorithm.

### 3.1 Optimization formulation

Given a target structure $y^{*}$, the following standard constrained optimization is adopted to find suitable RNA sequence *x*, where *f* can be equilibrium probability or ensemble defect,


(10) $$\underset{x}{\text{minimize}}\;f(x,y^{*})\text{subject to}\;y^{*}\in Y(x)$$

(11) $$\begin{array}{cc} f(x,y^{*}) & =1-p(y^{*}|x),\;\;\text{or}\\ f(x,y^{*}) & =\text{NED}(x,y^{*}). \end{array}$$

Note that our work is a general approach, therefore either of two ensemble objectives can work. Although we will see in the experiments that choosing equilibrium probability as objective is able to solve more puzzles and get more MFE solutions, we believe the actual choice between the two really depends on the application scenario and the biological perspective of experts.

### 3.2 Multifrontier search framework

Inspired by beam search, a popular heuristic to prune the search space in computational linguistics, which keeps top *k* highest-scoring results when parsing a natural language sentences (Huang and Chiang 2005), we develop a multifrontier search framework for RNA design. The optimization for Equation (10) starts with an initialization stage, i.e. a number (*k*) of sequences are initialized using our targeted initialization rules. Then an iterative process was repeated until the end. During the iterative search process, a priority queue is used to store the top *k* RNA sequences with the lowest objectives in the history of all iterations. At each of the iterative step, there are three stages:

1. Sampling: First one sequence is selected from the sequences in the priority queue, then a position is sampled from the selected sequences for mutation.
2. Mutation: The sequence selected in sampling stage is mutated by structured mutation based on targeted structure and the sampled position.
3. Updating: The mutated sequence is evaluated with objective function and the MFE/uMFE criterion, then the priority queue and MFE/uMFE solutions are updated.

Compared to random walk based approaches (Hofacker et al. 1994; Zadeh et al. 2011; Yang et al. 2017; Portela 2018), our work keeps best-*k* results rather than just best-1 result at each step. The reason comes from that there is an incompatibility between our ensemble objective and MFE criterion, i.e. the successfully designed sequence by MFE criterion may not be the one with lowest objective value and vice versa. Therefore, it is necessary to keep multiple suboptimal sequences rather than only one at each iteration.

Compared to genetic algorithm-based approaches (Taneda 2011; Lyngsø et al. 2012; Kleinkauf et al. 2015; Rubio-Largo et al. 2019), our work does not utilize the crossover operator to generate new sequences. The reason is that the actual RNA folding engines are nonlinear and very complicated, therefore there is no guarantee that the crossover operator can generate a sequence with enough affinity to their parents. In contrast, we adopt the mutation operator because it only changes a few nucleotides at a time, therefore the resulted new sequence is ensured to be very close to the old one.

### 3.3 Targeted initialization

Given a target structure $y^{*}$, the choice of the initial sequence $x^{\left(0\right)}$ is very important. Previous work has used random initialization according to databases (Bellaousov et al. 2018), energy (Busch and Backofen 2006), weighted sampling (Reinharz et al. 2013) or completely random choice of starting sequence. However, we choose an extremely simple scheme: for each unpaired position *i* in the target structure, we set it to be $x_{i}^{\left(0\right)}=A$, and for each base pair (*i*, *j*) in the target structure, we set the two nucleotides $\left(x_{i}^{\left(0\right)},x_{j}^{\left(0\right)}\right)$ to be either (G,C) or (C,G) at random. This scheme makes sure the unpaired positions will not pair with paired positions, and the paired positions are matched, but it does not rule out unwanted base pairs, i.e. we want (*i*, *j*) to pair and in the MFE structure (or ensemble), *i* pairs (or tends to pair) with some other paired position j′. However, the random choice between GC and CG pairs for paired positions is actually quite effective when there are relatively long stacking helices due to complementarity, one example is shown below.

12345678901234567890

(((...)))..(((...)))

CGGAAACCGAAGCGAAACGC

We speculate that the effectiveness may have to do with GC stacks being the most stable one in the Turner model (Turner and Mathews 2010a). In our initialization scheme, the two sides of each helix are guaranteed to be complementary to each other ($x_{\left[1:3\right]}^{\left(0\right)}$ with $x_{\left[7:9\right]}^{\left(0\right)}$, and $x_{\left[12:14\right]}^{\left(0\right)}$ with $x_{\left[18:20\right]}^{\left(0\right)}$), but the $x_{\left[1:3\right]}^{\left(0\right)}$ substring is chosen randomly over ${\left\{C,G\right\}}^{3}$, so it is highly unlikely that it can be complementary with another helix, in this case with either $x_{\left[12:14\right]}^{\left(0\right)}$ or $x_{\left[18:20\right]}^{\left(0\right)}$. In fact, for a helix of *h* stacked pairs, the likelihood of finding a fully complementary sequence is $1/2^{h}$. Given that stacking energy (especially the stacking of (G,C) or (C,G)) is the most important feature in the standard RNA folding energy model, long complementary spans tend to pair with each other, so it is likely that the target structure is favored in the ensemble. To kick off the search with multifrontier, we repeat such targeted initialization until *k* different sequences $\left\{x^{\left(0:1\right)},x^{\left(0:2\right)},\ldots ,x^{\left(0:k\right)}\right\}$ are found.

### 3.4 Boltzmann sampling

The sampling stage can be split into two. The first sampling is to select the most promising sequence from the priority queue. Suppose the top *k* sequences at step *t* are $\left\{x^{\left(t:1\right)},x^{\left(t:2\right)},\ldots ,x^{\left(t:k\right)}\right\}$, we set the probability of sequence $x^{\left(t:l\right)}$ being selected for mutation as the following *Boltzmann distribution* where *T* is the parameter to control the flatness of the distribution. As T →0, the distribution will approach Dirac (“one-hot”) distribution; as T →∞, the distribution will approach uniform distribution.


(12) $$p^{\left(t:l\right)}=\frac{\;\text{exp}\;\left((1-f(x^{\left(t:l\right)},y^{*}))/T\right)}{\sum_{m=1}^{k}\;\text{exp}\;\left((1-f(x^{\left(t:m\right)},y^{*}))/T\right)}\;.$$

The second sampling is position sampling, which intends to select the most critical position to mutate. To give more chances to the improperly paired position in the ensemble, the positions are sampled based on positional ensemble defect. Suppose the selected sequence at step *t* is $x^{\left(t\right)}$, the probability of position *i* being chosen for mutation is set from another *Boltzmann distribution* where *N* is the length of target structure $y^{*}$ and T′ is the parameter to control the flatness of the distribution.


(13) $$p_{i}^{\left(t\right)}=\frac{e^{\epsilon_{i}(x^{\left(t\right)},y^{*})/T\prime }}{\sum_{j=1}^{N}e^{\epsilon_{j}(x^{\left(t\right)},y^{*})/T\prime }}\;,$$

While it is flexible to have two temperatures T′ and *T* for two distributions separately, we found that the performance of SAMFEO is not sensitive to *T*. To simplify our model, we set T′=2T.

### 3.5 Structured mutation

**Figure 1.**
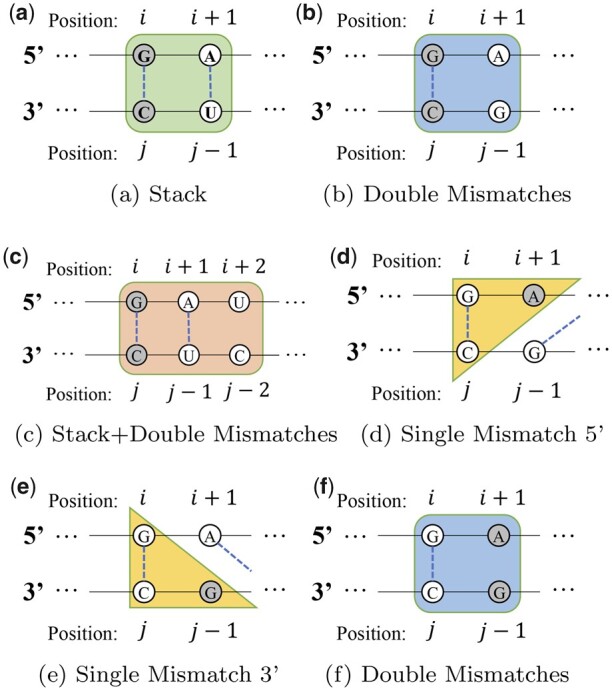
Diagrams of structured mutation. Paired positions are connected by blue dashed lines and each of the rounded rectangles or triangles represents a specific local structure. Diagrams a, b, c show structured mutation with paired positions (shaded nucleotides pair). Diagrams d, e, f show structured mutation with unpaired positions (shaded nucleotides). When a shaded position is selected for mutation, all the positions within the same local structure would be mutated simutaneously.

The widely used mutation method exchanges either one base at the selected position or one base pair containing the selected position. Such change is the minimal mutation which can guarantee the target structure is still in the ensemble of mutated sequence. However, in this way, the physical model of RNA’s secondary structure is totally ignored. In fact, neither a single nucleotide nor a base pair can form an atomic unit contributing to the global free energy in popular RNA fold engines such as Vienna (Lorenz et al. 2011), LinearFold (Huang et al. 2019) CONTRAfold (Do et al. 2006), and RNAstructure (Reuter and Mathews 2010). For paired positions, they are often evaluated with adjacent paired positions or unpaired positions, which forms the basic unit of stacking or mismatch. Similarly, unpaired positions are often evaluated with adjacent paired positions, which constitutes the sequence-dependent terms that stabilize or destabilize loop sequence in the Turner energy model (Turner and Mathews 2010b). Based on such observations, we propose to mutate the sequence in a way such that the sampled position would be mutated together with other positions within the same local structures such as stacking and mismatch. The visualization of structured mutation is illustrated in Fig. 1. We describe the mutation operator for paired and unpaired positions separately. For paired positions, our proposed mutation operator involves three scenarios: stack, double mismatches, and stack plus double mismatches.

1. **Stack** (diagram 1a). When either *i* or *j* of the stacking pair positions (i,j),(i+1,j−1) in the target structure is sampled, the two pairs are mutated as a whole. This approach offers 6×6−1=35 possibilities in total for one mutation, given that each pair has six choices in {CG,GC,AU,UA,GU,UG}.
2. **Double mismatches** (diagram 1b). When the paired positions (*i*, *j*) are next to two mismatched positions *i* + 1 and *j* − 1, and *i* or *j* of the pair positions (*i*, *j*) in the target structure is sampled, the paired positions (*i*, *j*) and unpaired positions(i+1,j−1) are mutated simultaneously, which results in 6×4×4−1=95 possible mutations, given that each pair has six choices and each unpaired position has four choices from {A,C,G,U}.
3. **Stack plus double mismatches** (diagram 1c).The situation with both (1) and (2) are also handled. If either *i* or *j* of the stacking pair positions (i,j),(i+1,j−1) in the target structure is sampled, and the positions *i* + 2, *j* − 2 are mismatched, the pair positions (i,j),(i+1,j−1), and two mismatched positions *i* + 2, *j* − 2 are mutated at the same time. This approach offers 6×6×4×4−1=575 possibilities in total, given that each pair has six choices, and each unpaired position has four choices.

Similarly, for unpaired positions, our proposed mutation operator involves three scenarios: single mismatch on the side of the 5′ end, single mismatch on the side of the 3′ end, and double mismatches.

1. **Single mismatch on the side of** 5′ **end** (diagram 1d). For the paired positions (*i*, *j*), if position *i* + 1 is unpaired and position *j* − 1 is paired, when position *i* + 1 is sampled for mutation, position *i* + 1 and paired positions (*i*, *j*) are mutated together, offering 4×6−1=23 possible mutations.
2. **Single mismatch on the side of** 3′ **end** (diagram 1e). For the paired positions (*i*, *j*), if position *i* + 1 is paired and position *j* − 1 is unpaired, when the position j−1 is sampled for mutation, position j−1 and paired positions (*i*, *j*) are mutated together, resulting in 4×6−1=23 possible mutations.
3. **Double mismatches** (diagram 1f). For the paired positions (*i*, *j*), if both position *i* + 1 and position *j* − 1 are unpaired, when *i* + 1 or *j* − 1 is sampled for mutation, the unpaired positions *i* + 1, *j* − 1 and paired positions (*i*, *j*) are mutated together, offering 4×4×6−1=95 possible mutations.

To handle the cases where the selected position is in a long unpaired region, we apply a trivial mutation, which simply changes the nucleotide at the selected position to a random nucleotide. Although this type of mutation does not take into account the local structures of RNA, it still provides a certain degree of diversity in the generated sequences. In summary, the use of structured mutation expands the search space, as it involves more changes in one step compared to traditional mutation methods. This can be beneficial in avoiding local minima during optimization. However, the increased search space may require more iterations to fully explore. To balance effectiveness and efficiency, we have selected the most critical (Anderson-Lee et al. 2016) local structures to incorporate structured mutation into the optimization process.

### 3.6 Updating with byproducts

Each new sequence is evaluated with the objective function and two categories of results are updated. First, the priority queue, i.e. the multifrontier, will be updated based on the objective value of the new sequence. This operation is quite efficient using the standard “enqueue” operation in the data structures implemented in modern programming languages. Second, because SAMFEO optimizes either equilibrium probability or ensemble defect, the sequence with lowest objective value does not have to be a MFE solution. Instead of optimizing multiple objectives, we propose to treat MFE solutions as the byproducts of optimizing our single ensemble objective. Specifically, we check the MFE/uMFE criterion for each new sequence along the way of optimization. As long as the new sequence generated by structured mutation at each step satisfies the MFE/uMFE criterion, it will be saved as one of the designed sequences at the end.

### 3.7 Pseudocode and complexity analysis

Algorithm 1: SAMFEO

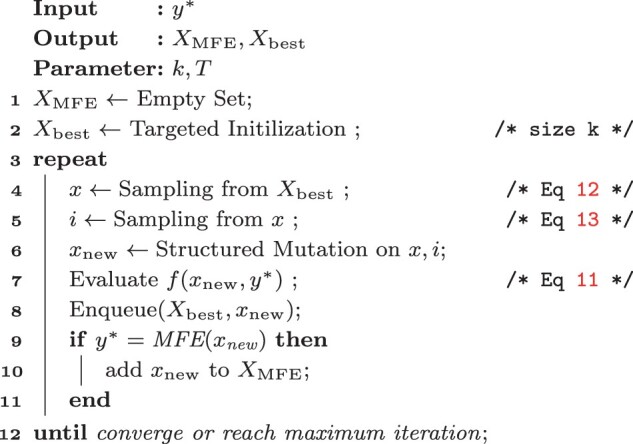

The pseudocode of SAMFEO is presented in Algorithm 1. The input is a target structure $y^{*}$ of length *n* and output consists of a set of MFE solutions $X_{\text{MFE}}$ and a set of sequences $X_{\text{best}}$ which have the lowest objectives in optimization process. Target initialization (line 2) is used to generate the initial *k* sequences. Inside the iterations, two samplings (line 4 and 5) are performed first, which are followed by Structured Mutation (line 6). Then the mutated sequence $x_{\text{new}}$ is evaluated by the objective function (line 7). The objective value of $x_{\text{new}}$ will decide whether it will get into the priority queue (line 8). Finally, the MFE criterion will be checked, which once is satisfied $x_{\text{new}}$ will be kept as a MFE solution (lines 9–11). During this process, the most time consuming steps are the evaluation of objective function and check of MFE criterion, either of which takes $O(n^{3})$ times. The time cost of other steps such as initialization and the maintenance of priority queue is negligible in contrast. Therefore, when the maximum number of iterations (denoted as *M*) is fixed, the complexity of SAMFEO is $O(n^{3})$. When *M* is also regarded as a part of the input, the complexity will be $O(Mn^{3})$.

## 4 Experiment

First, we compare the performance of SAMFEO against other approaches on the well-known benchmark Eterna100. Then, we show the superiority of SAMFEO over the baselines on long sequences design. Finally, an ablation study is conducted to demonstrate the effectiveness of the ingredients of SAMFEO.

### 4.1 Evaluation setting

We selected the following RNA design methods as baselines.

1. RNAinverse, the default RNA design algorithm in ViennaRNA package 2.0 (Lorenz et al. 2011).
2. RNAinverse-pf, which is another version of RNAinverse optimizing equilibrium probability.
3. NUPACK 4.0 (Zadeh et al. 2011), which utilizes hierarchical decomposition to optimize ensemble defect.
4. MODENA (Taneda 2011), which uses genetic algorithm to optimize structure distance and minimum free energy.
5. NEMO (Portela 2018), which combines Nested Monte Carlo Search with domain-specific knowledge for RNA design.
6. m2dRNAs (Rubio-Largo et al. 2019), which applies genetic algorithm to optimize three objectives at the same time.

Those baselines are widely used in the literature (Garcia-Martin et al. 2013; Anderson-Lee et al. 2016; Portela 2018; Rubio-Largo et al. 2019). Note that the primary goal of this work is to design a specific secondary structure, therefore RNA design methods focusing on other constraints are not considered here. The options of baselines are mostly set as default. The specific options can be seen in Supplementary Section 3.

By default, our method SAMFEO and all the baselines except NUPACK use the folding engine from ViennaRNA package 2.0. To make a more sensible comparison, we particularly implemented a version of SAMFEO using the folding algorithm from NUPACK and compare it with the design method in NUPACK package, which is presented in Supplementary Section 1.1. We run all programs on Linux, with 3.40 GHz Intel Xeon E3-1231 CPU and 32G memory. We set the default parameters of SAMFEO as *k *=* *10, *T *=* *1.0, and *M *=* *5000. The convergence condition in Algorithm 1 is set as when the objective value of $f(x,y^{*})$ is smaller than 0.01 or can’t get better for 2000 consecutive iterations.

### 4.2 Design Eterna100

#### 4.2.1 Overall metric

We evaluate the performance of SAMFEO and baselines with the widely used benchmark Eterna100 (Anderson-Lee et al. 2016), which contains a total of 100 secondary structure design challenges that span a large range in design difficulty, from short hairpins to complex 400-nucleotide designs. Following the settings in the literature (Anderson-Lee et al. 2016; Rubio-Largo et al. 2019), each method is run five times to design a puzzle, we report either union or average metrics accordingly. Except for extra explanation, the following metrics are used for evaluation:

1. Number of solved puzzles by MFE solutions. We report both union and average value over 5 runs.
2. Number of solved puzzles by Unique MFE (uMFE) solutions. (We use the command “RNAsubopt -e 0” in ViennaRNA package to get all MFE structures.) We report both union and average value over 5 runs.
3. Number of MFE solutions per solved puzzles with MFE solutions. We report the union value over 5 runs.
4. Number of uMFE solutions per solved puzzles with uMFE solutions. We report the union value over 5 runs.
5. Average equilibrium probability over all puzzles, i.e. $p(y^{*}|x)$, where *x* is the designed sequence and $y^{*}$ is the target structure. We report the average value over 5 runs.
6. Average ensemble defect over all puzzles, i.e. $\text{NED}(x,y^{*})$, where *x* is the designed sequence and $y^{*}$ is the target structure. We report the average value over 5 runs.
7. Positional entropy (Garcia-Martin and Clote 2015), which measures the entropy of all the possible pairs in the ensemble. We report the average value over 5 runs.

The comparable metrics on Eterna100 are shown in Table 1. We implemented SAMFEO with two different objectives Prob (equilibrium probability) and NED (ensemble defect) separately. SAMFEO solves 77 and 74 puzzles by MFE and uMFE criterions respectively, outperforming all the other methods except NEMO. However, it turns out such slight advantage of NEMO does not come from its Nested Monte Carlo Search but from the coded heuristic rules (specialized to the ViennaRNA energy model) acquired by personal experience without performing computational optimizations. We tested the vanilla version of NEMO, from which the heuristic rules are ablated, and its design results are presented in Supplementary Section 1.2, showing that the vanilla version of NEMO solves 76 puzzles, less than SAMFEO.

**Table 1.** Metrics of different RNA design methods on solving the 100 puzzles of Eterna100.^a^

Method	Objective	Union (5 runs)		Average (5 runs)		Union (5 runs)		Average (5 runs)		
		Solved puzzles↑		Solved puzzles↑		Solutions/solved.puzzle↑		Prob ↑	NED↓	PosEntropy↓
		MFE	uMFE	MFE	uMFE	MFE	uMFE			
RNAinverse	BPD	30	27	18.0	13.0	3.0	2.4	0.039	0.402	0.878
RNAinverse-pf	Prob	70	70	62.8	62.8	4.5	4.5	0.503	0.069	0.163
NUPACK	NED	34	34	27.8	27.6	4.1	4.1	0.170	0.098	0.074
MODENA	Multi-2	64	64	60.6	60.2	165.9	144.6	0.198	0.147	0.320
NEMO	Comp	**79**	**77**	**76.8**	66.8	4.8	4.3	0.178	0.146	0.339
m2dRNAs	Multi-3	72	70	70.4	66.2	48.1	47.9	0.302	0.138	0.316
SAMFEO	NED	72	66	66.2	62.8	9865.7	10334.6	0.493	**0.042**	**0.054**
	Prob	77	74	73.4	**70.4**	**13498.7**	**13408.7**	**0.559**	0.061	0.117

a Note on abbreviations: BPD: base pair distance is the number of base pairs that occur in exactly one of the two compared structures (Ward et al. 2022), i.e. $\text{BPD}(y,y^{*})=|\mathrm{pairs}(y)\cup \mathrm{pairs}(y^{*})|-|\mathrm{pairs}(y)\cap \mathrm{pairs}(y^{*})|$. Multi-2/3: multiple objectives (Taneda 2011; Rubio-Largo et al. 2019); Comp: a composite function of base pair distance and free energy difference(ΔΔG), i.e. $1-\frac{\text{BPD}(y,y^{*})}{2|\mathrm{pairs}(y*)|}$ and $\frac{1}{1+\Delta \Delta G}$ (Portela 2018); PosEntropy: positional entropy. Note on fonts: bold: the best value in its column. Underline: the second best value in its column.

When we use the average metric, SAMFEO can solve the most number (70.4) of puzzles for each run by uMFE criterion. Most importantly, the average equilibrium probability of the target structures can reach almost 0.56 by SAMFEO, which means most of the structures in the ensemble of designed sequences would be the same as target structures. This result of equilibrium probability is better than RNAinverse-pf (a relative improvement of over 10%), which demonstrates the superiority of SAMFEO (Prob) since they are optimizing the same objective. Similarly, SAMFEO with NED as objective can achieve the lowest ensemble defect as well as the lowest positional entropy, which support our claim that SAMFEO is a general optimization paradigm. Another striking observation is that SAMFEO can generate a large quantity of MFE solutions, which is much more than all the other methods including the genetic algorithm MODENA and m2dRNAs.

#### 4.2.2 MFE study

**Figure 2.**
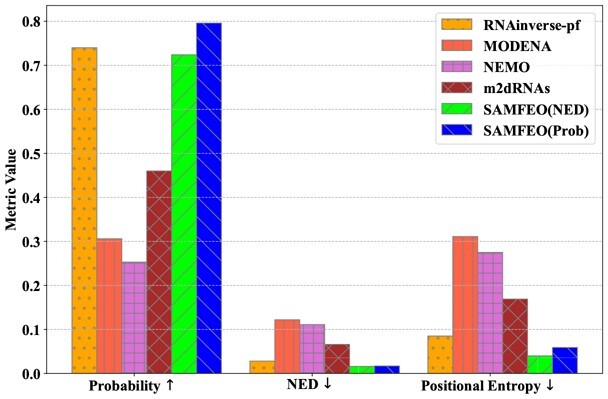
Ensemble metrics of 64 solved puzzles in Eterna100.

**Figure 3.**
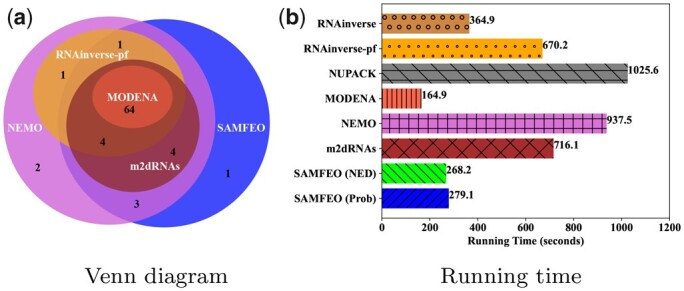
Results of MFE study and efficiency study. (a) shows the Venn diagram of solved puzzles by different methods. (b) shows the running time of different methods to design Eterna100 puzzles.

We investigate the quality of the MFE solutions found by each method. First, we plot the Venn diagram of methods solving more than 60 puzzles in Fig. 3a. Nearly all the puzzles solved by MODENA, RNAinverse-pf and m2dRNAs fall into the intersection of NEMO and SAMFEO, which contains 64 puzzles. To get more insight into the quality of MFE solutions by different methods, we show the average ensemble metrics of MFE solutions for the 64 puzzles in particular, which is presented in Fig. 2. All the values are average over 5 runs. As we can see from the bar plot, either SAMFEO (Prob) or SAMFEO (NED) has lower NED and Positional entropy than other four strong baselines. In terms of equilibrium probability, SAMFEO (Prob) is the best with an average probability close to 0.8 while SAMFEO (NED) is slightly lower than RNAinverse but also reaches a probability over 0.7. Therefore, we demonstrate that our proposal SAMFEO find the MFE solutions of higher quality than others. For a more detailed plot, including standard deviations as error bars, please see Supplementary Section 2.1. In addition, the diversity of MFE solutions are shown in Supplementary Section 5.1.

#### 4.2.3 Efficiency study

Figure 3b shows the average running time that different methods spend on solving each puzzle. We observe that SAMFEO is also very efficient and faster than most baselines. In contrast, the two strong baselines m2dRNAs and NEMO need much more time than SAMFEO. For a more detailed plot, including error bars indicating standard deviation, please see Supplementary Section 2.2.

**Figure 4.**
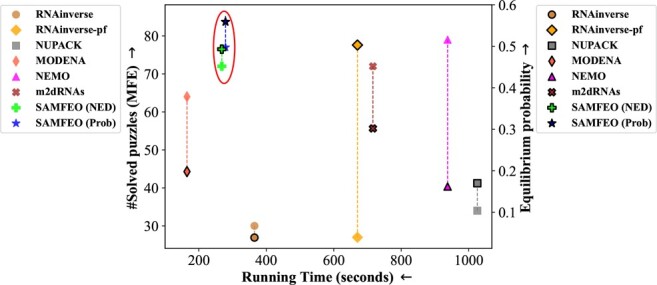
MFE and equilibrium probability versus time for Eterna100 design. The red ellipse highlights our proposed methods.

We also plot the scatter of number of solved puzzles versus running time and the scatter of equilibrium probability vs. running time in Fig. 4. The numerical values of running time are taken from Fig. 3b and the number of solved puzzles and the values of equilibrium probability are taken from Table 1. We can see that SAMFEO appears at the top left corner with respect to either metric, which illustrates that SAMFEO achieves an excellent balance between efficacy and efficiency.

### 4.3 Long sequence design

Although the Eterna100 benchmark spans a large range in design features and difficulty to validate the ability of RNA design, the lengths of puzzles are all less than 400. To further test the ability of various RNA design methods to design long secondary structures, we select 10 sequences of length over 900 from 16S Ribosomal RNAs dataset and use the MFE structures folded by ViennaRNA package 2.0 as puzzles. The specific sequences and structures are included in Supplementary Sections 6.2 and 6.3, respectively. For brevity, we detail the design result of five puzzles from one time design by each baseline and SAMFEO (Prob), which is shown in Table 2. The corresponding RNA sequences designed by SAMFEO are presented in Supplementary Section 6.4. Specifically, for each of the five puzzles, we report the metrics of one sequence designed by each method. For the methods outputting multiple MFE solutions, we select the most probable one. See Supplementary Section 6.1 for the full result of running each method five times to design the puzzles. See Supplementary Section 5.2 for the similarity between the designed RNAs and the original 16S Ribosomal RNAs.

**Table 2.** Long sequences design results. ✓/✗ represent whether the designed sequence satisfies MFE or uMFE criterion.

Method	Length = 1200			Length = 1452			Length = 1474			Length = 1490			Length = 1492		
	Prob↑	MFE	uMFE	Prob ↑	MFE	uMFE	Prob↑	MFE	uMFE	Prob↑	MFE	uMFE	Prob↑	MFE	uMFE
RNAinverse	1.6e-28	✗	✗	4.2e-44	✗	✗	7.0e-37	✗	✗	5.3e-34	✗	✗	7.7e-41	✗	✗
RNAinverse-pf	3.7e-05	✗	✗	7.7e-18	✗	✗	8.5e-16	✗	✗	2.2e-12	✗	✗	4.8e-30	✗	✗
NUPACK	5.2e-04	✗	✗	7.9e-14	✗	✗	9.4e-16	✗	✗	2.2e-16	✗	✗	4.1e-12	✗	✗
MODENA	1.7e-04	**✓**	✗	3.4e-09	✗	✗	8.8e-06	**✓**	**✓**	5.5e-08	✗	✗	2.4e-07	✗	✗
NEMO	6.4e-07	**✓**	✗	9.7e-12	**✓**	✗	2.4e-09	**✓**	✗	2.5e-10	**✓**	**✓**	3.0e-10	**✓**	✗
m2dRNAs	2.8e-04	**✓**	✗	1.5e-05	**✓**	✗	1.6e-03	**✓**	**✓**	4.2e-04	**✓**	**✓**	2.3e-03	**✓**	**✓**
SAMFEO	**0.242**	**✓**	**✓**	**0.031**	**✓**	**✓**	**0.246**	**✓**	**✓**	**0.185**	**✓**	**✓**	**0.209**	**✓**	**✓**

As we can see in Table 2, the baselines perform much worse than when designing Eterna100. RNAinverse and NUPACK do not find any MFE solutions for those long puzzles while MODENA can only solve two puzzles by MFE criterion. Even though NEMO and m2dRNAs can solve all puzzles by MFE criterion, not all their design satisfy uMFE criterion. By comparison, SAMFEO solves all the five long puzzles by both MFE and uMFE criterion. The most striking observation comes from the fact that the equilibrium probabilities of other methods’ design are as low as nearly zero, regardless of MFE criterion satisfaction. In contrast, the designs of SAMFEO exhibit a much higher equilibrium probability in the ensemble. Therefore, SAMFEO shows great advantages over other methods for designing long sequences.

### 4.4 Ablation study

To validate the effectiveness of different components of our proposal, we performed ablation study for the performance of SAMFEO (Prob) on Eterna100 design. Specifically, following versions of SAMFEO are experimented.

1. SAMFEO-v1, Targeted Initialization (TI) is ablated, and random base pairs are used for initialization.
2. SAMFEO-v2, Boltzmann Sampling (BS) is ablated. Instead Samplings are performed randomly.
3. SAMFEO-v3, Structured Mutation (SM) is ablated and traditional mutation is adopted.
4. SAMFEO-v4, Multi-Frontier (MF) is ablated. We set *k* = 1 such that there only keeps one sequence in the priority queue.
5. SAMFEO-v5, The way of MFE solutions as byproducts (BP) is ablated (only the sequence with lowest objective is used).

We run each version five times and the results are shown in Table 3. We can see that any ablation of Targeted Initialization, Boltzmann Sampling, Structured Mutation, or MFE solutions as byproducts leads to less solved puzzles. Compared to SAMFEO, SAMFEO-v4 even solves one more puzzle by union metrics. However, the numbers of MFE and uMFE solutions per solved puzzles reduce from over 13 400 to around 11 200 and 11 000, respectively. Such a drop might be detrimental for RNA design when we need diverse MFE solutions in two cases: (i) RNA design with more constraints, and (ii) obtaining MFE solutions for learning-based RNA design. Finally, it is at the discretion of users to determine the parameters of the RNA design algorithm according to real world application.

**Table 3.** Ablation study on Eterna100.

Method	Ablation	Union (5 runs)		Average (5 runs)		Union (5 runs)		Average (5 runs)		
		Solved puzzles↑		Solved puzzles↑		Solutions/solved.puzzle↑		Prob ↑	NED↓	PosEntropy↓
		MFE	uMFE	MFE	uMFE	MFE	uMFE			
SAMFEO	None	77	74	73.4	70.4	13 498.7	13408.7	0.559	0.061	0.117
SAMFEO-v1	TI	70	67	65.2	62.4	12 161.3	12087.6	0.510	0.133	0.184
SAMFEO-v2	BS	74	71	71.4	68.2	13 734.2	13786.3	0.558	0.061	0.114
SAMFEO-v3	SM	74	72	70.4	68.0	15 237.1	15152.0	0.550	0.059	0.109
SAMFEO-v4	MF	78	75	73.4	70.4	11 207.2	11066.6	0.559	0.060	0.108
SAMFEO-v5	BP	75	73	70.6	68.0	3.9	4.3	0.559	0.061	0.117

## 5 Conclusion and future work

We propose a new RNA design paradigm, which optimizes single ensemble objective and generate MFE and uMFE solutions as byproducts. By utilizing both structure level and ensemble level information during iterative multifrontier search process, our algorithm SAMFEO can perform effective and efficient design for the benchmark Eterna100. In addition, SAMFEO presents a huge advantage over other design methods in terms of long sequences design. Our work has several potential extensions.

1. Constrained RNA design. In the literature (Garcia-Martin et al. 2013; Esmaili-Taheri and Ganjtabesh 2015), there are RNA design scenarios where other constraints are imposed.
2. Learning to design. Since our work can find a large amount of MFE and uMFE solutions, it is helpful to generate more training data to boost the effect of learning-based RNA design.
3. Hierarchical decomposition. Our work evaluates the objective on the whole sequence, which might not be efficient enough for very long sequences, it would save a lot of time if useful structure or sequence decomposition can be used.

## Supplementary Material

btad252_Supplementary_Data
