# Supplementary material for: RNA design via structure-aware multifrontier ensemble optimization
Source: Bioinformatics. 2023 Jun 30;39(Suppl 1):i563–71. doi: 10.1093/bioinformatics/btad252 (PMC10311297; doi:10.1093/bioinformatics/btad252)
Supplement: btad252_Supplementary_Data [file btad252_supplementary_data.pdf]

ISMB, 2023, 1–10
doi: DOI HERE
Advance Access Publication Date: Day Month Year
Paper

Supplementary Data: RNA Design via Structure-Aware Multi-Frontier Ensemble Optimization

Tianshuo Zhou, Ning Dai, Sizhen Li, Max Ward, David H. Mathews and Liang Huang

Abstract

This is the supplementary material for the paper SAMFEO published at ISMB2023.

1. Design Eterna100

1.1. SAMFEO using folding engine of NUPACK

The result is shown in Table 1.

1.2. NEMO without heuristic rules

The result is shown in Table 2.

2. Bar plot with deviation

2.1. MFE study

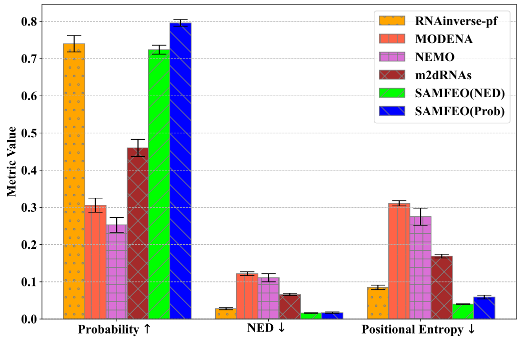

Fig. 1: Ensemble metrics of 64 solved puzzles in Eterna100.

2.2. Efficiency study

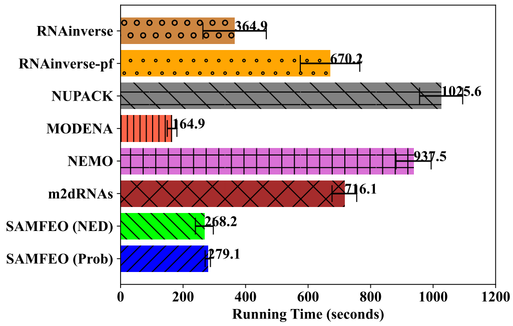

Fig. 2: Running time of Eterna100 design by different methods.

3. Settings of Baselines

Eterna100 design.

1. RNAinverse: default.
2. RNAinverse-pf: default.
3. NUPACK: default.
4. MODENA: -pop 50; -step 100.
5. NEMO: default.
6. m2dRNAs: 5000 iterations with time limit of 3600 seconds.

Long sequences design.

1. RNAinverse: default.
2. RNAinverse-pf: default.
3. NUPACK: default.
4. MODENA: -pop 50; -step 200.
5. NEMO: default.
6. m2dRNAs: 2000 iterations with time limit of 10000 seconds.

© The Author 2023. Published by Oxford University Press. All rights reserved. For permissions, please e-mail: journals.permissions@oup.com

Table 1. Metrics of different RNA design methods on solving the 100 puzzles of Eterna100.

Method	Objective	Union (5 runs) Solved puzzles↑		Average (5 runs) Solved puzzles↑		Union (5 runs) Solutions / solved.puzzle↑		Average (5 runs)		
		MFE	uMFE	MFE	uMFE	MFE	uMFE	Prob ↑	NED↓	PosEntropy↓
NUPACK	NED	34	34	27.8	27.6	4.1	4.1	0.170	0.098	0.074
SAMFEO	NED	48	47	42.8	42.0	2082.9	2061.0	0.290	0.090	0.051
	Prob	51	50	49.8	48.4	10200.0	10026.9	0.386	0.105	0.141

Table 2. Metrics of different RNA design methods on solving the 100 puzzles of Eterna100.

Method	Objective	Union (5 runs) Solved puzzles↑		Average (5 runs) Solved puzzles↑		Union (5 runs) Solutions / solved.puzzle↑		Average (5 runs)		
		MFE	uMFE	MFE	uMFE	MFE	uMFE	Prob ↑	NED↓	PosEntropy↓
NEMO	Comp	76	75	76.0	67.4	4.0	4.5	0.162	0.147	0.335
SAMFEO	NED	72	66	66.2	62.8	9865.7	10334.6	0.493	0.042	0.054
	Prob	77	74	73.4	70.4	13498.7	13408.7	0.559	0.061	0.117

4. RNAinverse with Targeted Initialization

We applied our proposed initialization method to RNAinverse and RNAinverse-pf, the results are shown in Table 3 and Table 4.

5. Diversity and Identity

5.1. Diversity of MFE solutions

We calculate the nucleotide diversity of each position by measuring the entropy of the distribution on $\{A, C, G, U\}$. A higher entropy value indicates a more diverse nucleotide composition at that position. In Table 5, we present the average entropy over all positions for a set of MFE solutions, obtained from 64 puzzles (of Eterna100) that can be solved by most methods.

5.2. Sequence Identity

To assess the similarity between the designed RNAs and the original 16s rRNAs used to generate the dataset of long sequences, we calculate the proportion of equal nucleotides at each position to obtain both the maximum and minimum sequence identity for a set of MFE solutions. We present the results obtained from NEMO, m2dRNAs, and SAMFEO in Table 6, as they are capable of solving all 10 long puzzles.

Table 3. Metrics of different RNA design methods on solving the 100 puzzles of Eterna100. "TI" means "Targeted Initilization" in the table.

Method	Objective	Union (5 runs) Solved puzzles↑		Average (5 runs) Solved puzzles↑		Union (5 runs) Solutions / solved.puzzle↑		Average (5 runs)		
		MFE	uMFE	MFE	uMFE	MFE	uMFE	Prob ↑	NED↓	PosEntropy↓
RNAinverse	BPD	30	27	18.0	13.0	3.0	2.4	0.039	0.402	0.878
RNAinverse+TI	BPD	66	65	59.6	57.4	4.4	4.3	0.285	0.114	0.245
RNAinverse-pf	Prob	70	70	62.8	62.8	4.5	4.5	0.503	0.069	0.163
RNAinverse-pf+TI	Prob	74	69	69.4	66.0	4.4	4.5	0.546	0.050	0.113
SAMFEO	NED	72	66	66.2	62.8	9865.7	10334.6	0.493	0.042	0.054
	Prob	77	74	73.4	70.4	13498.7	13408.7	0.559	0.061	0.117

Table 4. Metrics of different RNA design methods on solving the 10 puzzles adapted from 16S. "TI" means "Targeted Initilization" in the table.

Method	Objective	Union (5 runs) Solved puzzles↑		Average (5 runs) Solved puzzles↑		Union (5 runs) Solutions / solved.puzzle↑		Average (5 runs)		
		MFE	uMFE	MFE	uMFE	MFE	uMFE	Prob ↑	NED↓	PosEntropy↓
RNAinverse	BPD	0	0	0	0	0.0	0.0	0.000+e00	0.433	0.989
RNAinverse+TI	BPD	9	9	9.0	6.8	5.0	3.8	0.012	0.012	0.038
RNAinverse-pf	Prob	2	2	1.6	1.6	4.0	2.5	7.820e-02	4.880e-02	1.174e-01
RNAinverse-pf+TI	Prob	2	2	2.0	2.0	5.0	5.0	0.111	0.015	0.033
SAMFEO	Prob	10	10	8.2	6.6	9483.3	6934.5	0.146	0.004	0.018

Table 5. Diversity of MFE solutions for 64 solved puzzles in Eterna100.

Method	Objective	Nucleotide Entropy ↑
RNAinverse-pf	Prob	0.439
MODENA	Multi-2	**0.735**
NEMO	Comp	0.623
m2dRNAs	Multi-3	0.462
SAMFEO	NED	0.608
	Prob	0.649

Table 6. Sequence identity between designed sequences and original 16s rRNAs.

Method	Objective	max. sequence identity	min. sequence identity
NEMO	Comp	0.335	0.301
m2dRNAs	Multi-3	0.372	0.347
SAMFEO	Prob	0.374	0.353

6. Long Sequence Design

6.1. Overall metrics

The result is shown in Table 7.

6.2. 16S Ribosomal RNAs

1. AAUAGGUUUGGUCCUAGCCUUUCUAUUAACUCUUAGUAGGAUUACACAUGCAA
GCAUCCCCGCCCCAGUGAGUCACCCUCUAAAUCACCACGAUCAAAAGGAACAA
GCAUCAAGUACGCAGAAAUGCAGCUCAAAACGCUUAGCCUAGCCACACCCCCA
CGGGAGACAGCAGUGAUAAACCUUUAGCAAUAAACGAAAGUUUAACUAAGCCA
UACUAACCCCAGGGUUGGUCAAUUUCGUGCCAGCCACCGCGGUCACACGAUUA
ACCCAAGCCAAUAGAAAUCGGCGUAAAGAGUGUUUUAGAUCAAUCCCCCAAUA
AAGCUAAAAUUCACCUGAGUUGUAAAAAACUCCAGCUGAUAUAAAAUAAACUA
CGAAAGUGGCUUUAAUAUAUCUGAACACACAAUAGCUAGGACCCAAACUGGGA
UUAGAUACCCCACUAUGCCUAGCCCUAAACUUCAACAGUUAAAUUAACAAGAC
UGCUCGCCAGAACACUACGAGCCACAGCUUAAAACUCAAAGGACCUGGCGGUG
CUUCACAUCCUUCUAGAGGAGCCUGUUCUGUAAUCGAUAAACCCCGAUCAACC
UCACCACCUCUUGCUCAGCCUAUAUACCGCCAUCUUCAGCAAACCCUGACGAA
GGCCACAAAGUAAGCACAAGUACCCACGUAAAGACGUUAGGUCAAGGUGUAGC
CCAUGAGGUGGCAAGAAAUGGGCUACAUUUUCUACUUCAGAAAACUACGAUAA
CCCUUAUGAAACCUAAGGGUAGAAGGUGGAUUUAGCAGUAAACUAAGAGUAGA
GUGCUUAGUUGAACAGGGCCCUGAAGCGCGUACACACCGCCCGUCACCCUCCU
CAAGUAUACUUCAAAGGACAUUUAACUAAAACCCCUACGCAUCUAUAUAGAGG
AGAUAAGUCGUAACAUGGUAAGUGUACUGGAAAGUGCACUUGGACGAAC
2. AAUAGGUUUGGUCCUAGCCUUUCUAUUAGCUCUUAGUAAGAUUACACAUGCAA
GCAUCCCCGUUCCAGUGAGUUCACCCUCUAAAUCACCACGAUCAAAAGGAACA
AGCAUCAAGCACGCAGCAAUGCAGCUCAAAACGCUUAGCCUAGCCACACCCCC
ACGGGAAACAGCAGUGAUUAACCUUUAGCAAUAAACGAAAGUUUAACUAAGCU
AUACUAACCCCAGGGUUGGUCAAUUUCGUGCCAGCCACCGCGGUCACACGAUU
AACCCAAGUCAAUAGAACCCGGCGUAAAGAGUGUUUUAGAUCACCCCCUCCCC
AAUAAAGCUAAAACUCACCUGAGUUGUAAAAAACUCCAGUUGACACAAAAUAG
ACUACGAAAGUGGCUUUAACAUAUCUGAACACAGAAUAGCUAAGACCCAAACU
GGGAUUAGAUACCCCACUAUGCUUAGCCCUAAACCUCAACAGUUAAAUCAACA
AAACUGCUCGCCAGAACACUACGAGCCACAGCUUAAAACUCAAAGGACCUGGC
GGUGCUUCAUAUCCCUCUAGAGGAGCCUGUUCUGUAAUCGAUAAACCCCGAUC
AACCUCACCACCUCUUGCUCAGCCUAUAUACCGCCAUCUUCAGCAAACCCUGA
UGAAGGCUACAAAGUAAGCGCAAGUACCCACGUAAAGACGUUAGGUCAAGGUG
UAGCCCAUGAGGUGGCAAGAAAUGGGCUACAUUUUCUACCCCAGAAAACUACG
AUAGCCCUUAUGAAACUUAAGGGUCGAAGGUGGAUUUAGCAGUAAACUAAGAG
UAGAGUGCUUAGUUGAACAGGGCCCUGAAGCGCGUACACACCGCCCGUCACCC
UCCUCAAGUAUACUUCAAAGGACAUUUAACUAAAACCCCUACGCAUUUAUAUA
GAGGAGACAAGUCGUAACAUGGUAAGUGUACUGGAAAGUGCACUUGGACGAAC
3. AAAUUAGAGUUUGGUGCUGGCUCAGCUUUCAUGCGCCGAGCGGUGGUUUAUAC
AUGCGAGCUUAAAAGAUUAAGGCCACGAGUGCGUAAUACGCGAGUUUAAAUUU
AUUAUACAAAAGUUGCUAGGUGACCCAAUACGGGCAUCGGGCAAAACUCGCGU
AGGAUUAGCGAGCUGGGUCGCGACGCCAAUACAGCGGCCCAUGGCUUAUCCUU
AGCCUGUCUUAACGGUACUAGGCCACGGUGGCACUGAAAAGGGGCCACGGUUC
UUAUGAACCCAGCAGUGUUGAAUUUUGGACAAUCGCUUGAACGGCGGAUCCAG
AUGCUGCUUACAAAAUUGUUUGUGAACACCCCCCAAGCACGUGCCAGAAGGGU
CGGUAAAACGUGCGGUGUCAGUAUAAAGCGUCUUGACUAGGCAGGCAGCGCGU
CUGAGCGUUAAUGGUGAACACUUUAAACGUUGGGUAAUAUUCGGAGGAUCGGU
CAAAUGAGAAUAUUCCGGAUGGAAAGCCGAAGGCGAAAGCACCAACAUCAGAG
UCACUAAAGCUUCAACGCGUAAGUUUGGGUAGCGAACCGGAUUAGAGACCCGC
AGUAGUCCAAACCGUCAACACAUUAUAGUUUUCAAAUCUAUAACGCCUGGUGA
UACGGUGGCAACACUAUAAAUCAAAGCAAUUGGCAGCGAUAGAGAUGCGCGGU
GGAAUAUGCUGUUUAAAUCGAAUUUACGCGCAAAAUCUUACCACUUUUUAUUA
AAAUAGCUCUUGCAUUGCUGAAAAGUUCGAGCGGGACCUCGCCCUUAGUGCCG
GUCUUUGCCACGUUUUCUGAACGGUCGAGCAAGUCAUCAUGGGGCUUAUAGAG
UGGGCUACAGGCGUAUUACAUUGGACACCCACAAGUUGGAUACCAAAACUGUC
CGAAUAUACGGAUUGGAGUAGCAAAACGAGAGAACUAUACAGUUCGUUUGCAU
CGUUAUGCCUUGCUGAAACUAGCCUCCAUGAAGAAGGAAUCGCGAGUAAUCGU
AGAUCAUUAGCGCUACGGUGAAGGUAACCUCUAUUGUGCACACAUUGCCCGUC
ACCUCCGAUAAUAGUAUUGUACAGGAAGAACUAUGGCUACACUUAAGUCGCGG
CCUAGGAACGUAUGCGUGAUAUUAGAGUUGGAGUAAGUCGUAACAGGUUGGGG
UAGGGGAACCUGCUCCAGAGUCAAAUUUAUAUAA
4. CAUCCGGUCGAUCCUGCCGGAGCGCGACGCUCUCCCCAAGGACGAAGCCAUGC
AUGCCCGCUCACCCGGGACGCGGCGGACGGCUCAGGACAACGGUUGCACCCCC
CGCGGCGGUCCCUGCUAGCCGGACACCGCUGGCAACCCGGCGCCAAGACGUGC
GCGCAAGGGCGGGCGCCCGCGGGCGAGCAGCGUGACGCAGCGACGGCCCGCCC
GGGCUUCCGGGGCAUCACCCGGUCGGCGCGGUCGCGGCGCGCCGAGGGCCCGA
CGCCUGGCGGAGAAUCAGGGUUCGACUCCGGAGAGCGGGCCUGCGAGACGGCC
CGCACAUCCAAGGACGGCAGCAGGCGCGGAACUUGCCCAAUGCGCGGCGCGCG
AGGCAGCGACGGGGAGCGCGCGAGCGAGGCGGGCCCACAGCCCCCGCCGCGGA
GCCGAGGGCAAGGUCUGGUGCCAGCAGCCGCGGUAAUUCCAGCUCGGCGAGCG
UCGCGCGGCGCUGCUGCAGUUGAAACGCCCGUAGUUGGCCCCCCGCCGCCACG
AGGAAACGGGAGCGCUCCAGGCAGGCCCGUUGGACCCGCCGCGUGGGACCGCG
CAGCGGGCCGGCGCGCCGCGGCAGCCCCGAGGAGAGCGGGCGGGGGCACCGGU
ACCGGCCGGGGACGGGUGAAACAGGAUGAUCCCGCCGAGACCGCCGGCCGCGC
AGGCGCCUGCCAAGACCGCCUCUGUCAAUCAAGGGCGAAGGCCGGGGGCUAGA
AGGCGAUCAGACACCACCGUAUUCCCGGCCGUAAACGGUGCCGCCCCGCGGCC
GGCGCGCGCGUCCCGCCGGCCGCCCAGGGAAACCGGGAGGCUCCGGGCUCUGG
GGGGAGUAUGGCCGCAAGGCUGAAACUUGAAGGCAUUGACGGAGGGGUACCAC
CAGACGUGGAGUCUGCGGCUCAAUCUGACUCAACGCGCGCACCUCACCAGGCC
CGGACGCGCGGAGGACCGACAGCCGGGCGCGCUUUCGCGAUCGCGCGGGCGGU
GGUGCAUGGCCGCUCCCAGCCCGUGGCGCGAGCCGUCUGCUCCAUUGCGACAA
CGAGCGAGACCCCGGCCGCGGGCGCCGCGGGACGGCCCGCGCGAGCGGGAGGA
CGGCGGGGCGAUAGCAGGUCUGUGAUGCCCUCAGACGCCCUGGGCCGCACGCG
CGCUACACUGGCGGGGCCAGCCGGCGCGCGCGAGGACGCGCGGAGCCCCCGCC
GUGGCCGGGACCGCGGGCUGGAACGCCCCCGCGCACCAGGAAUGUCUUGUAGG
CGCCCGCCCCCACCGCGCGCCGGACGCGUCCCUGCCCCUUGUACACACCGCCC
GUCGCUCCUACCGACUGGGCGCGGCGGCGAGCGCCCCGGACGCGCGAAGGGCC
GCGAGCCCCCGCGCCUGGAGGAAGGAGAAGUCGUAACAAGGUAUUCCGUAGGU
GAACCUGCGGAUGGAUCCCUC
5. AUCCAUGGAGAGUUUGAUCCUGGCUCAGGACGAACGCUGGCGGCAUGCUUAAC
ACAUGCAAGUCGAACGAGCAAAGCAAUUUGUGUAGUGGCGAACGGGUGCGUAA
CGCGUAAGAACCUACCUAUCGGAGGGGGAUAACAUUGGGAAACUGUUGCUAAU
ACCCCAUACAGCUGAGGAGUGAAAGGUGAAAAACCGCCGAUAGAGGGGCUUGC
GUCUGAUUAGCUAGUUGGUGGGGGUAACGGCCUCCCAAGGCCACGAGCAGUAG
CUGGUCUGAGAGGAUGAUCAGCCACACUGGGACUGAGACACGGCCCAGACUCC
UACGGGAGGCAGCAGUGAGGAAUUUUUCGCAAUGGGCGCAAGCGACGGAGCAA
UGCCGCGUGCAGGAAGAAGGCCUGUGGGUCGUAAACUGCUUUUCUCAGAGAAG
AAGUUCUGACGGUAUCUGAGGAAUAAGCACCGGCUAACUCUGUGCCAGCAGCC
GCGGUAAUACAGAGGGUGCAAGCGUUGUCCGGAAUGAUUGGGCGUAAAGCGUC
UGUAGGUGGCUCGUAAAGUCUAAUGUCAAAUACCAGGGCUCAACCUUGGACCG
GCAUUGGAGUACUCACGAGCUUGAGUACGGUAGGGGCAGAGGGAAUUCCAUGU
GGAGCGGUGAAAUGCGUAGAGAUAUGGAGGAACACCAGUGGCGAAGGCGCUCU
GCUGGGCCGAAACUGACACUGAGAGACGAAAGCUGGGGGAGCGAAUAGGAUUA
GAUACCCUAGUAGUCCCAGCCGUAAACUAUGGAGACUAAGUGCUGCCGCAAGC
AGUGCUGUAGCUAACGCGUUAAGUCUCCCGCCUGGGGAGUAUGCUCGCAAGAG
UGAAACUCAAAGGAAUUGACGGGACCGCACAAGCGGUGGAUUAUGUGGAUUAA
UUCGAUACAACGCGAAGAACCUUACCAGGGUUUGACAUGUCAAGAACCUCUCA
GAAAUGGGAGGGUGCCCUAACGGACUUGAACACAGGUGGUGCAUGGCUGUCGU
CAGCUCGUGCUGUGAAGUGUAUAGUUAAGUCUCAUAACGAGCGCAACCCUCGU
CUUUAGUUGCCAUUUGGUUCUCUAAAGAGACUGCCAGUGUAAGCUGGAGGAAG
GUGAGGAUGACGUCAAGUCAGCAUGCCCCUUACAUCCUGGGCUUCACACGUAA
UACAAUGGUUGGGACAAUCAGAAGCGACUCGUGAGAGCUAGCGGCUCUGUUAA
ACCCAACCUCAGUUCGGAUUGUAGGCUGCAACUCGCCUACAUGAAGCCGGAAU
CGCUAGUAAUCGCCAGUCAGCUAUAUGGCGGUGAAUACGUUCCCGGGUCUUGU
ACACACCGCCCGUCACACCAUGGAAGCUGGUUCUGCUCCAAGUCGUUACCCUA
ACCUUCGGGAGGGGGGCGCCUAAAGCAGGGCUAGUGACUAGGGUGAAGUCGUA
ACAAGGUAGGGCUACUGGAAGGUGGCCCUGGCUCACCUCCUUC

Table 7. Metrics of different RNA design methods on solving 10 puzzles adapted from 16S.

Method	Objective	Union (5 runs) Solved puzzles↑		Average (5 runs) Solved puzzles↑		Union (5 runs) Solutions / solved.puzzle↑		Average (5 runs)		
		MFE	uMFE	MFE	uMFE	MFE	uMFE	Prob ↑	NED↓	PosEntropy↓
RNAinverse	BPD	0	0	0	0	0.0	0.0	0.000+e00	0.433	0.989
RNAinverse-pf	Prob	2	2	1.6	1.6	2.5	2.5	7.820e-02	4.880e-02	1.174e-01
NUPACK	NED	0	0	0.0	0.0	0.0	0.0	0.000e+00	1.840e-02	4.820e-02
MODENA	Multi-2	5	2	3.2	2.0	4.0	4.5	2.000e-04	5.620e-02	1.628e-01
NEMO	Comp	10	5	10.0	1.4	5.0	1.4	0.000+e00	6.080e-02	1.584e-01
m2dRNAs	Multi-3	10	8	7.6	4.8	3.2	2.5	0.006	0.017	0.050
SAMFEO	Prob	10	10	8.2	6.6	9483.3	6934.5	0.146	0.004	0.018

6. AUUCCGGUUGAUCCUGCCGGAGGUCAUUGCUAUUGGGGUCCGAUUUAGCCAUG
CUAGUUGCACGAGUUCAUACUCGUGGCGAAAAGCUCAGUAACACGUGGCCAAA
CUACCCUACAGAGAACGAUAACCUCGGGAAACUGAGGCUAAUAGUUCAUACGG
GAGUCAUGCUGGAAUGCCGACUCCCCGAAACGCUCAGGCGCUGUAGGAUGUGG
CUGCGGCCGAUUAGGUAGACGGUGGGGUAACGGCCCACCGUGCCGAUAAUCGG
UACGGGUUGUGAGAGCAAGAGCCCGGAGACGGAAUCUGAGACAAGAUUCCGGG
CCCUACGGGGCGCAGCAGGCGCGAAACCUUUACACUGCACGCAAGUGCGAUAA
GGGGACCCCAAGUGCGAGGGCAUAUAGUCCUCGCUUUUCUCGACCGUAAGGCG
GUCGAGGAAUAAGAGCUGGGCAAGACCGGUGCCAGCCGCCGCGGUAAUACCGG
CAGCUCAAGUGAUGACCGAUAUUAUUGGGCCUAAAGCGUCCGUAGCCGGCCAC
GAAGGUUCAUCGGGAAAUCCGCCAGCUCAACUGGCGGGCGUCCGGUGAAAACC
ACGUGGCUUGGGACCGGAAGGCUCGAGGGGUACGUCCGGGGUAGGAGUGAAAU
CCCGUAAUCCUGGACGGACCACCGAUGGCGAAAGCACCUCGAGAAGACGGAUC
CGACGGUGAGGGACGAAAGCUAGGGUCUCGAACCGGAUUAGAUACCCGGGUAG
UCCUAGCUGUAAACGAUGCUCGCUAGGUGUGACACAGGCUACGAGCCUGUGUU
GUGCCGUAGGGAAGCCGAGAAGCGAGCCGCCUGGGAAGUACGUCCGCAAGGAU
GAAACUUAAAGGAAUUGGCGGGGGAGCACUACAACCGGAGGAGCCUGCGGUUU
AAUUGGACUCAACGCCGGACAUCUCACCAGCUCCGACUACAGUGAUGACGAUC
AGGUUGAUGACCUUAUCACGACGCUGUAGAGAGGAGGUGCAUGGCCGCCGUCA
GCUCGUACCGUGAGGCGUCCUGUUAAGUCAGGCAACGAGCGAGACCCGCACUU
CUAAUUGCCAGCAGCAGUUUCGACUGGCUGGGUACAUUAGAAGGACUGCCGCU
GCUAAAGCGGAGGAAGGAACGGGCAACGGUAGGUCAGUAUGCCCCGAAUGAGC
UGGGCUACACGCGGGCUACAAUGGUCGAGACAAUGGGUUGCUAUCUCGAAAGA
GAACGCUAAUCUCCUAAACUCGAUCGUAGUUCGGAUUGAGGGCUGAAACUCGC
CCUCAUGAAGCUGGAUUCGGUAGUAAUCGCAUUUCAAUAGAGUGCGGUGAAUA
CGUCCCUGCUCCUUGCACACACCGCCCGUCAAAGCACCCGAGUGAGGUCCGGA
UGAGGCCACCACACGGUGGUCGAAUCUGGGCUUCGCAAGGGGGCUUAAGUCGU
AACAAGGUAGCCGUAGGGGAAUCUGCGGCUGGAUCACCUCCUG
7. CUCAUGGAGAGUUCGAUCCUGGCUCAGGAUGAACGCUGGCGGCAUGCUUAACA
CAUGCAAGUCGAACGGGAAGUGGUGUUUCCAGUGGCGAACGGGUGAGUAACGC
GUAAGAACCUGCCCUUGGGAGGGGAACAACAACUGGAAACGGUUGCUAAUACC
CCGUAGGCUGAGGAGCAAAAGGAGAAAUCCGCCCAAGGAGGGGCUCGCGUCUG
AUUAGCUAGUUGGUGAGGCAAUAGCUUACCAAGGCGAUGAUCAGUAGCUGGUC
CGAGAGGAUGAUCAGCCACACUGGGACUGAGACACGGCCCAGACUCCUACGGG
AGGCAGCAGUGGGGAAUUUUCCGCAAUGGGCGAAAGCCUGACGGAGCAAUGCC
GCGUGGAGGUGGAAGGCCUACGGGUCGUCAACUUCUUUUCUCGGAGAAGAAAC
AAUGACGGUAUCUGAGGAAUAAGCAUCGGCUAACUCUGUGCCAGCAGCCGCGG
UAAGACAGAGGAUGCAAGCGUUAUCCGGAAUGAUUGGGCGUAAAGCGUCUGUA
GGUGGCUUUUCAAGUCCGCCGUCAAAUCCCAGGGCUCAACCCUGGACAGGCGG
UGGAAACUACCAAGCUGGAGUACGGUAGGGGCAGAGGGAAUUUCCGGUGGAGC
GGUGAAAUGCAUUGAGAUCGGAAAGAACACCAACGGCGAAAGCACUCUGCUGG
GCCGACACUGACACUGAGAGACGAAAGCUAGGGGAGCAAAUGGGAUUAGAGAC
CCCAGUAGUCCUAGCCGUAAACGAUGGAUACUAGGUGCUGUGCGACUCGACCC
GUGCAGUGCUGUAGCUAACGCGUUAAGUAUCCCGCCUGGGGAGUACGUUCGCA
AGAAUGAAACUCAAAGGAAUUGACGGGGGCCCGCACAAGCGGUGGAGCAUGUG
GUUUAAUUCGAUGCAAGGCGAAGAACCUUACCAGGGCUUGACAUGCCGCGAAU
CCUCUUGAAAGAGAGGGGUGCCCUCGGGAACGCGGACACAGGUGGUGCAUGGC
UGUCGUCAGCUCGUGCCGUAAGGUGUUGGGUUAAGUCUCGCAACGAGCGCAAC
CCUCGUGUUUAGUUGCCACUAUGAGUUUGGAACCCUGAACAGACCGCCGGUGU
UAAGCCGGAGGAAGGAGAGGAUGAGGCCAAGUCAUCAUGCCCCUUAUGCCCUG
GGCGACACACGUGCUACAAUGGGCGGGACAAAGGGUCGCGAUCUCGCGAGGGU
GAGCUAACUCCAAAAACCCGUCCUCAGUUCGGAUUGCAGGCUGCAACUCGCCU
GCAUGAAGCAGGAAUCGCUAGUAAUCGCCGGUCAGCCAUACGGCGGCGAAUCC
GUUCCCGGGCCUUGUACACACCGCCCGUCACACUAUAGGAGCUGGCCAGGUUU
GAAGUCAUUACCCUUAACCGUAAGGAGGGGGAUGCCUAAGGCUAGGCUUGCGA
CUGGAGUGAAGUCGUAACAAGGUAGCCGUACUGGAAGGUGCGGCUGGAUCACC
UCCUUU
8. AUUCUGGUUGAUCCUGCCAGAGGCCGCUGCUAUCCGGCUGGGACUAAGCCAUG
CGAGUCAAGGGGCUUGUAUCCCUUCGGGGAUGCAAGCACCGGCGGACGGCUCA
GUAACACGUGGACAACCUGCCCUCGGGUGGGGGAUAACCCCGGGAAACUGGGG
CUAAUCCCCCAUAGGGGAUGGGUACUGGAAUGUCCCAUCUCCGAAAGCGCUUA
GCGCCCGAGGAUGGGUCUGCGGCGGAUUAGGUUGUUGGUGGGGUAACGGCCCA
CCAAGCCGAAGAUCCGUACGGGCCAUGAGAGUGGGAGCCCGGAGAUGGACCCU
GAGACACGGGUCCAGGCCCUACGGGGCGCAGCAGGCGCGAAACCUCCGCAAUG
CGGGAAACCGCGACGGGGUCAGCCGGAGUGCUCGCGCAUCGCGCGGGCUGUCG
GGGUGCCUAAAAAGCACCCCACAGCAAGGGCCGGGCAAGGCCGGUGGCAGCCG
CCGCGGUAAUACCGGCGGCCCGAGUGGCGGCCACUUUUAUUGGGCCUAAAGCG
UCCGUAGCCGGGCUGGUAAGUCCUCCGGGAAAUCUGGCGGCUUAACCGUCAGA
CUGCCGGAGGAUACUGCCAGCCUAGGGACCGGGAGAGGCCGGGGGUAUUCCCG
GAGUAGGGGUGAAAUCCUGUAAUCCCGGGAGGACCACCUGUGGCGAAGGCGCC
CGGCUGGAACGGGUCCGACGGUGAGGGACGAAGGCCAGGGGAGCGAACCGGAU
UAGAUACCCGGGUAGUCCUGGCUGUAAACGAUGCGGACUAGGUGUCACCGAAG
CUACGAGCUUCGGUGGUGCCGGAGGGAAGCCGUUAAGUCCGCCGCCUGGGGAG
UACGGCCGCAAGGCUGAAACUUAAAGGAAUUGGCGGGGGAGCACUACAACGGG
UGGAGCCUGCGGUUUAAUUGGAUUCAACGCCGGGAAGCUUACCGGGGGAGACA
GCGGGAUGAAGGUCGGGCUGAAGACCUUACCAGACUAGCUGAGAGGUGGUGCA
UGGCCGCCGUCAGUUCGUACUGUGAAGCAUCCUGUUAAGUCAGGCAACGAGCG
AGACCCGCGCCCCCAGUUGCCAGCGGUUCCCUUCGGGGAAGCCGGGCACACUG
GGGGGACUGCCGGCGCUAAGCCGGAGGAAGGUGCGGGCAACGGCAGGUCCGUA
UGCCCCGAAUCCCCCGGGCUACACGCGGGCUACAAUGGCCGGGACAAUGGGUA
CCGACCCCGAAAGGGGUAGGUAAUCCCCUAAACCCGGUCUAACCUGGGAUCGA
GGGCUGCAACUCGCCCUCGUGAACCUGGAAUCCGUAGUAAUCGCGCCUCAAAA
UGGCGCGGUGAAUACGUCCCUGCUCCUUGCACACACCGCCCGUCAAGCCACCC
GAGUGGGCCAGGGGCGAGGGGGUGGCCCUAGGCCACCUUCGAGCCCAGGUCC
GCGAGGGGGGCUAAGUCGUAACAAGGUAGCCGUAGGGGAAUCUGCGGCUGGAU
CACCUCCU
9. ACUCCGGUUGAUCCUGCCGGGCCCGACCGCUAUCGGGGUGGGACUAAGCCAUG
GGAGUCGUGCGCCCCCGGACGCGGGGGCGCGGCGGACGGCUGAGUAACACGUG
GCCAACCUACCCUCGGGACGGGGAUAACCCCGGGAAACUGGGGCUAAUCCCCG
AUAGGCGAGGGGGCCUGGAACGGGUCCCUCGCCGAAAGGGCGCGCGGAGCCUC
CCCGCGCGCCGCCCGAGGAUGGGGCUGCGGCCCAUCAGGUAGUUGGCGGGGUA
ACGGCCCGCCAAGCCGAUAACGGGUAGGGGCCGUGAGAGCGGGAGCCCCCAGA
UGGGCACUGAGACAAGGGCCCAGGCCCUACGGGGCGCACCAGGCGCGAAACCU
CCGCAAUGCGGGCAACCGUGACGGGGUCACCCCGAGUGCCGCCGAUAAGGCGG

CUGUUCCCCGCUGUAGGAAGGCGGGGGAGUAAGCGGGGGGCAAGUCUGGUGUC
AGCCGCCGCGGUAAUACCAGCCCCGCGAGCGGUCGGGAUGAUUACUGGGCCUA
AAGCGCCCGUAGCCGGCCCGGUAAGUCCCCCCUAAAGCCCCGGGCUCAACCC
GGGGAGUGGGGGGGAUACUGCCGGGCUAGGGGGCGGGAGAGGCCGAGGGUACU
CCCGGGGUAGGGGCGAAAUCCGAUAAUCCCGGGAGGACCACCAGUGGCGAAGG
CGCUCGGCUGGAACGCGCCCGACGGUGAGGGGCGAAAGCCGGGGGAGCAAACC
GGAUUAGAUACCCGGGUAGUCCCGGCUGUAAACGAUGCGGGCUAGGUGUUGGG
CGGGCUUGGAGCCCGCCCAGUGCCGCAGGGAAGCCGUUAAGCCCGCCGCCUGG
GGAGUACGGCCGCAAGGCUGAAACUUAAAGGAAUUGGCGGGGGAGCACCACAA
GGGGUGGAGCCUGCGGCUUAAUUGGAGUCAACGCCGGGAAUCUUACCGGGGGC
GACAGCAGGAUGACGGCCAGGCUGACGACCUUGCCCGACACGCUGAGAGGAGG
UGCAUGGCCGUCGCCAGCUCGUGCCGUGAGGUGUCCGGUUAAGUCCGGCAACG
AGCGAGACCCCCACCCCUAGUUGCUACCCCGGGGCCACCCCGGGGGCACACUA
GGGGGACUGCCGCCGUCCAAGGCGGAGGAAGGAGGGGGCCACGGCAGGUCAGC
AUGCCCCGAAUCCCCCGGGCUGCACGCGGGCUACAAUGGCGGGGACAGCGGGA
UCCGACCCCGAAAGGGGGAGGCAAUCCCUUAAACCCCGCCGCAGUUGGGAUCG
AGGGCUGCAACUCGCCCUCGUGAACGCGGAAUCCCUAGUAACCGCGCGUUAGC
AUCGCGCGGUGAAUACGUCCCUGCUCCUUGCACACACCGCCCGUCGCUCCACC
CGAGCGGGGGAGGGGUGAGGCCCCGUCCCUCGCCAGGGCGGGGUCGAACCUCU
CCCCCGCGAGGGGGGAGAAGUCGUAACAAGGUAGCCGUAGGGGAACCUGCGGC
UGGAUCACCUCCU

10. CAAUUGAAGAGUUUGAUCCUGGCUCAGAAUGAACGUUGGCGGCAUGGAUUAGG
CAUGCAAGUCGUGCGCGAUAUGUAGCAAUACAUGGAGAGCGGCGAAAGGGAGA
GUAAUACGUAGGAACCUACCUUCGGGUCUGGGAUAGCGGCGGGAAACUGCCGG
UAAUACCAGAUGAUGUUUCCGAACCAAAGGUGUGAUUCCGCCUGAAGAGGGGC
CUACGUCGUAUUAGCUAGUUGGUAGGGUAAUGGCCUACCAAGGCAAAGAUGCG
UAUGGGGUGUGAGAGCAUGCCCCCACUCACUGGGACUGAGACACUGCCCAGAC
ACCUACGGGUGGCUGCAGUCGAGAAUCUUCGGCAAUGGGCGAAAGCCUGACCG
AGCGAUGCCGCGUGCGGGAUGAAGGCCUUCGGGUUGUAAACCGCUGUCGUAGG
GGAUGAAGUGCUAGGGGGUUCUCCCUCUAGUUUGAGCUGAACCUAGGAGGAAG
GGCCGGCUAAUCUCGUGCCAGCAGCCGCGGUAAUACGAGAGGCCCAAACGUUA
UUCGGAUUUACUGGGCUUAAAGAGUUCGUAGGCGGUCUUGUAAGUGGGGUGUG
AAAUCCCUCGGCUCAACCGAGGAACUGCGCUCCAAACUACAAGACUUGAGGGG
GAUAGAGGUAAGCGGAACUGAUGGUGGAGCGGUGAAAUGCGUUGAUAUCAUCA
GGAACACCGGAGGCGAAGGCGGCUUACUGGGUCCUUUCUGACGCUGAGGAACG
AAAGCUAGGGGAGCAAACGGGAUUAGAUACCCCGGUAGUCCUAGCCGUAAACG
AUGAGCACUGGACCGGAGCUCUGCACAGGGUUUCGGUCGUAGCGAAAGUGUUA
AGUGCUCCGCCUGGGGAGUAUGGUCGCAAGGCUGAAACUCAAAGGAAUUGACG
GGGGCUCACACAAGCGGUGGAGGAUGUGGCUUAAUUCGAGGCUACGCGAAGAA
CCUUAUCCUAGUCUUGACAUGCUUAGGAAUCUUCCUGAAAGGGAGGAGUGCUC
GCAAGAGAGCCUUUGCACAGGUGCUGCAUGGCUGUCGUCAGCUCGUGUCGUGA
GAUGUCGGGUUAAGUCCCUUAACGAGCGAAACCCUUGUCCUUAGUUACCAGCG
CGUCAUGGCGGGGACUCUAAGGAGACUGCCGGUGUUAAACCGGAGGAAGGUGG
GGAUGACGUCAAGUCCUCAUGGCCUUUAUGAUUAGGGCUGCACACGUCCUACA
AUGGUGCACACAAAGCGACGCAAACUCGUGAGAGCCAGCUAAUCGCAAAAAAU
GUACCUCAGUUCGGAUUGCAGGCUGCAACUCGCCUGCAUGAAGCUGGAAUCGC
UAGUAAUCGCGGGUCAGCAUACCGCGGUGAAUAUGUUCCUGAGCCUUGUACAC
ACCGCCCGUCAAGCCACGAAAGUGGGGGGGACCCAACAGCGCUGCCGUAACCG
CAAGGAACAAGGCGCCUAAGGUCAACUCCGUGAUUGGGACUAAGUCGUAACAA
GGUAGCCGUAGGGGAACCUGCGGCUGGAUCACCUCCUUUCU

6.3. Long Secondary Structures

1. ..((((...((((((((((..(((((((((...)))))))))...(((.(((..
...((((((.....(((......(((....(((.....)))....))).....
......(((..(((....))).)))......(((......)))..)))......
)))).))..)))))).......(((((...(((((....))))).)))))...
.(((((((....))))))).....(((((...(((.....)))..)))))...
......(((..........)))......(((((((((((..((.......(((
((((((....(((.(((((((......)))).))).)))............
......))))))))))..))))))).))))....))))))))).....(((((.
.(((.......)))..))))).))))((((.....)))).......((((..
((((..((((.(((((((.(...((.(((((.........(((..(((((((
....((.(.((((...(((..(((((((....((((........)))).....
.........................(((.((.((((......)))).)).
)))....((.(((((((..(.(((.((((....))))..)))).((((((((
((((............))))))))))))((((((.......((((....(
(((((((.......))))))).....))))..((((.......))))))))))
))))))).))))))))))..))))))).).))...))))))).)))...(((((
(..(((((......(((...............)))......))))).))))
)).))))).))....).)).))))).))))..))))..)))).......

2. ....((.((((..(((((((((...((.((((.((.(.(((((((((.((
(((((((.(.(((..((((((...(((....(((.....)))....)))....
.......(((..(((....))).))).......(((......)))....(((..
..)))....((((((........((((((..(((((....))))).(((((((
((.((((.((((((((((((.((......))))))))).(((.((((....((((
......))))....).))))))........(((.........)))........
.....(((((....(((.((((((((......)))).))).)))........(((
(((((....)))).))))).....((((....)))).)))))..........))
))).))))))).........))))))..................))))))......
..))))))...............((((....))))..))))))..))).).)).
))))))))))..........(((..((((((((....((((........)))).
.......(((((((((((...((.(((.....(((.((.((((......))))
.)).)))......))).))))))).(((.((((....))))..)))....(((
(((((((((..............))))))))))(((((.....)))))......
...(((((((.......)))))))...))))))(((.((((.(..(((...))
)..).)))).))).))))))))..)))...(((.((....)))))........(
(((((..(((((......(((..................)))........))))).
))))))....)).))))).)).)))).))..))))))).))....)))).)).

3. ((((((..((((((((((((.........))).)))))))))..))))))....
.(((((.((((.(((((..((((.(((....)))..((((((((((((......
............((((.((((.(((.....))))))).)))))))))))))))
)((((.(((.(..(((((((((............))))))))).))).))))..
.))))))))).((((((((((((....(((((.((.......))))))))(((((
....))))).....((((((((..((((((...(((((.....))))).)))))
)(((((((((((((.((..((((((.(((((......(((((((((.((.....)
))))...))))))))))))...)))))))..)).)))..)))))).)))((((((
..((((.....((((((...((((....(((((((..(((((((((..(((..
....))).....))))))))))....(((...)))....)).))))))...))))
))))))..))))..)))))))..((((((((..((....(((.........))).
.))..))))))))..))))))).((((((.........))))))).))))))))).
...((((....))))..........(((((((((((.((((((((((.((((((..
..........................)))))).........((((((((....
.(((.((....)))))..)))))))))......(..((((((((..((((((.(
((((((((((.(((((...)))))...).)))).((.(((((((((((...((..
(.(((...((((((((((...(((........)))....)))))).....(.(((
((......))))).)..(((((....((((.(((((....))))).))))....
.))))))))))).))).)..))))))).)))))).)).......((((....))))
)))))....))))))).))))).))))..)))))))))))))).)).)))))))))).
..((((((((..((((((((..((........(((.((((.(((....)).).))
)))))........))..)))))))))..))))))))))))))))).....((((((
((((....)))))))))))................

4. .....((..(((((((((.....((((((((((((((((..((.((((.....
..(((((((((.((((((..((((.(..((((((((((...((.((((((......
.))))).)).))))..)))))..).))))..((..((((...((....((((.
(((...(((.((((....(((((((((((((((..(((.((((((.(.((((.
(((((.((((((......)))))((((((((.......))))))))(((..(((
..((((((.......))))))..)))..)))(((..((((((((........))))
)))...)))...(.((((.((.((((((((.(((((((..(.((((((.(((.
..((.(((.(.....))))))..))).((.((((.....))))))))))))).)
.....))))))).))).)))))).)).)))))((.....))))))))))).)))
)))))))))))))))))..........))))))).....)))))))..)))))))

.))...)))).))(((((..((.(((.((((((.(((((.(((((....))))
).)))))))))))).)))))))).))))))).)).))))))))(((((((((..
..((((((((((..((((.....(..((((((((....((.((.((((((..((
((....))))......(((((.((.....)).)))))..)))))).))))...
.)).)))))..)..))))...))))))))))....))))))).))).(((((
((((.(......)))))))))))((.((....)).)).)))).))..)).)))
)))))))...(((.((((.......))))..)))..((((((((((((((....
..(((((((((..((((((...............)).)))).)))......(
(((.(((((((.((.......((((((..((((((((((((.(.((((((((
.(((((((((..(((...))))))).))))..))))))))).).)))))))...
.)))))....)))))).((((((((((((((((((.(((((....)))))...(
(((((((((.........(((((.(.....).))))))((((((.(((.(.((((
((((...(((((...)))))..))))))))).).)).).)).)).)))))))))
))..((.((..((((((..((....))))))))..)).))..)))))))).))
))))))))..)).)))))))))))).)))))))..)))))))...........))
))))(((((.((..((((((((((.(((..((((((((......))..))).)
))..))).))))))))))).)).))))))..))))).....)))).((((((((.
...)))))))).)))))))..

5. ......((((.(((((.(((((...))))))))))(((.(((((.(((.....
....))).)))))..).)))..........((((((((((..(((.((.....)
).)))..(..((((((((((.......))))...((((((..(((((((....
.(((((((((((((((((((((..(((.....)))(((........)))))))
.)))..))))))).))..))))))))))))))..))))))((((((..((((..(
((((.((((.........))))))...(((((.(((.....))))))))).((((
....))))......((((((....))))))..((.((.(((....((((.(((
(((.....((((......((((....)))).....)))).(((((((((....
..((....))....))))))))))...(((((......(((((((((.((....
)))))...))))))))))))..))))))))))).......))).)).)).)))..)
)))..)))))).......(((((((((.....((((((.....))))))...)
))))))))((((((((......)))))))))))))..)....(...((((((((
...(((.........)))....))))))))...).((((((((((....))))..
))))))))...))).)))).....((((...(((((((...(...........
)...))))))).(((((((((((.....((((.(((((((.(((((.(((...
..))).))))))....(((...(..(((((.....)))))..)...)))..(((
(...)))).........((((((((((((....)))))((((((.......)))
)))......(((.(..((((((((...))))))))..).)))......(((((((
....)))))))(((((.....((((((((((((.......((((((((((....
)))).))))))......))))....)))))))))......).))))....)))))
))))))))).))))).((((((((....))))..))))(((((((((..((((..
((((((.((((.....)))).......))))))).)))).)))))).))))....
....))))))))))))........((((((((.((((..((((((((.((((....
.((.(((....))).))..((((((((........))))))))......)))).)
))))))))..))))).))))).)))....(((((((..((((...........)))
)..))))))))))).((((.(((..((((((((((((...((.((((.((((.
.((...))..)))))))).))..))))))))))))..))).)))).......
...((.((((((((((....))))))))))).))...))))...

6. .....(((.(((((.(((.((((((((((((.((((((((((......(((.((
((.((..((((((((....))))))))((.....)).........((((((((..
....((((((((.((((..((.(((((((....))))))).))...))))....((
(((((..((......)).))))))))......(((....))))))))))...)))
)))))((((((((((((..(((((((((.......)))))))))))..)))))))
).(((((((.(........).))))))).....(((((((((......)))))))).(
(((....)))))))))))))))......((((.....(((((....)))))..))
))))))))))))..(((((((((.....)))))))))).((((((((((((((...)))
))))))))))...((((((.........((((((...((((...))))..))))))
))))))).)))))))))))........)).)))......(((((.(.((((((((((
(..(((((((((((...(((((((((.....)))))))))..)))))))))..)))
.))))))(((((.(((.....(((((((((((.((((((((((.(((......)
))....))))))))))).)))........((....)).))))))))....))).))
)))))))).).))))))..((((((((((.((....((((..........))))..))
))))))))....((((((((((((((.((((((((((((((((...))))))))))

)))))...((....))....)))))))..(((...((((.(((((....))))
)..))))..)))...((((((((((((((....(((((.((....)).))))).
.....((((....(((((.((.(((((((..(((...((((((((.((..(((.
(((((...))))).))).)).)))))))).)))..))))..((((((((((..
((((((.((.......(((((((.......))))..))).(((.....))).(((
(((((..(((((..((((....))))))))))....))))))))..((.(((((
.....))))))))...)).))))))).))))).)))))(((.((((.(......)
.))))))))...((((((((...(((((((.....(((((((..((((....))
))..)).))))).......)))))))))))))))...((((((((.........))
)))))(((((.(((((((....).)))).))))))))..))))).)))))....
.)))).)))))))............)))))))((((.(((..((((((((((((
(..((((((((....))))))))..))))))))))))))..))).)))..)))))
........(((((((((....)))))))))))))))))).....

7. ....((.(((((.((((((((..((((...(((((.((((((((........
))))).........((((((.......))))).((((((..((.((((((......((
(.......((((((((((((.((((.....((((((....)))))).......))
))....(((....)))....(((....))).))))))))..((((((.((..(((
((((((((..(((((((((.........))))))))))))..))))))))..)).))))
).......(((..((((((.(((((.(((.....)))))))))).((((....))
))......((((((....)))))))...((((....))))..((((..(((((.
((((((((((.((.((((....)))).)).)))))))(((((((((........
..........)))))))))..(((....)))..((((((((((.((....))))
)...)))))).))))..))))).))))........((((((...))))))...
.)))))))..)))..(((((((((.....((((((.....))))))...)))))
))))).(((.(((...))))))).((((...((((((((((..((((((((((....(
((((.....)))))..)))))))))).....))....((....)).)))))))..
))))...((((.((((((.(((....((((((((..((...((((.........
))))))..)))))))....((((.(((.(((..((((((((((((((........)
)))))))))))))))).)))...)))).....((((((((((..((((((((((((.
..........((((.....))))(.(((((.(((....)))((.((((.(.((
((....(((..(((...)))..)))....)))).))))).)).....(((..(
((((((....))))))).)))((((.((......(((((.((((((((((((((
((....)))).)))))).......((...(((((....((((((((((((((...
....)))))))).))))))......))).....)))))...))..)))))).))))
)...)).))))((((.(.(.((((((((...))).)))).)))))))..))))).
)))))...))))))).)..)))))))))).......(((((....)))))((((
(((....))).....))))))))))))))))))..((((((((.........))))
)))....))))))...)))......))))).))..))))).)).)))))).))))
((((((((((..((....))..))))))..........)))))).))))))).))
))).)).....(((((....)))))((((((..(((((....)))))((((((
((........))))))))).....((((((((((....))))))))))).....))
))))..

8. (((((((((((((((......((((((((..((((..((..(((.((((.(.
((......((.((((((((((((....)))))))))))).))..)).).)))).)
))..))..))))((((((((.((((((((((((((((..((((((....))))))
...)))))))))).((((((((((.((.......)))))))))))....((((...
)))))))))))).))))).))..(((((((.(((..((((((((........))))
))))))))...)))))))).((((((((....)))...))))).....(((((((.
.......))))))).((((....)))))))))).)))(((........)))...(
(((....)))).)))))))))))))))..((.(((((((..(..(((((.(
((((((........))))))))))))..).(((((.....((((.((((....)
))))))).....)))))((((((((((((...))))))).....)))))......((
(((.(.(((((((((((((.((((((((((.(..((((((((.....))))))))
.).))))))))))..))))))))...(((((.(....((((((.((.((((((
...((((((((....((((((.......))))))..))).)))))))).))).)).))
)))))....).))))))..)))).).))))))..((((((((..((...((((..
.......)))))))..)))))))))).....))))))))))...((((((((((((
((...)))))))))))))))((((((....(((((...((((((((((...(((
(.(((((....))))))..)))))..)))...))))))))..)))......(((((
(((((((((((((((..((((.........)))).)))))...(((((((((((
(((((((((((((((.........)))))))..((((((.(((((((..(((((((...

..)))))).)))))).)).))).....)))))))))..))).(((((((.(((
.....))).((((((((((((..((((((((....))).))))))))).))))
))))((((((((..(((..(((.......)))...)))..))))).)))))).
)))).....))))))))......)))))))).....(((((((.....(((((
((.(((((....))))).))))..))).....)))))))...((.((...(((
((((........))))))).....)).))....((((...((((((((......
.))))))))...))))....(((.....)))....))))))))..((((.(((
..(((((((..((((..((((((((((...))))))))))..))))..)))))
))..))).)))).................(((((((((....)))))))))))..
..))))).

9. ..(((((((..((((.((((.((((((((((..((((((((((((...((...
))))))).((((((((((....))))))))))((((.((((((.......((((
(((...(((.(((((((.(((((((..((((((....))))))...)))))))
...(((((((((.((......)).))))))))).....(((((((((......
.))))))))).))))))).))))))))))(((((((.(((..((((((((...
....))))))))))))))...)))).((((((.((....)).).)))))....
(((((.((......)).))))).((((....))))(((((((((......(((
((((...((((....))))..(((((...)))))...((((((.....)))))
)..(((((((((.......)))))))))....)))))))....))))))))))
)))))))))).......))).)))))..)))))))))).......((.(((((.
...(((((....(((((((((((.(((((((((.....(((((((.....)))
))))...)))))))))..))))))))).)))))))....))))).))(((.((
((((((((..(((.....)))....)))))))))).))).(((((((((....
))))..)))))..(((.(((.........)))))).....)))))))...))))
)))....(((.(((((....))))).)))..(((((((((((..(((((((((
((((((...))))))))))((((.(((((((.((((((.(((((((.((((((
((.((.(((..(((..(((((..........(((((((.(.((.((.((((..
...)))).)))).).)).))))))...(((..(.((((........)))).)..
)))(((.((.(((((((((.((..((..((((((.......))...))))..)
)))).))))))))))).)))(.(((((((..((..((((......))))..)).
.......(((((.(((((((((((..((((((((...)))))))))))).))))
))))..((.(((((......)))))))......))))).))))))).))))))
.))).))).)).))))))))......)))))))..))))))((((.....((((
(((..((((....))))..))..))))).....)))).((((.(((.(...((
(((((........)))))))).))))))))........((((((((((((....
..))))))))...))).))))))).....)))))))))))))))..((((.((
(..(((((((((((((..(((((((.((((....))))))))))..)))))))
)))))))..))).))))..)))))....((((((((((((((....)))))))
).....))))...

10. .....((((((..((((((.(((((((...((((((..((((((((((((((.
(...((..(((((((((.((((((....))))))((..(((((........((
((...(((((((..(((..((((((((((((..((.((((((....)))))).
))...))))..........((.(((...))).)).....)))))))))))..)
))))))((((((.(((..((((((((.......)))))))))))...))))))
...((((((((....)))))))).)))).(((((.(.........)))))).(
(((....)))))))))..))....((((((((...(((((....))))).)))
)).)))..)))))))))..))..(((((((((........))).(.((.((((
......((((((((((((....)))))))))....)))...)))).)).).))
))))).)))))).)))))).)).(((((((((.(((((((((((((...(((.
....)))....)))))))......))))))...(((((((((..((((((((.
.....((((((.....))))))....))))))))...)))))))))....(((
(((...(((((((((..((((((((.((((........))))..)))))))))
).....))....((....)).)))))))..))))))(((((.(((((((.((.
...(((((((..((...((((.........))))))..)))))))(((...((
.((((.(((.((((.((..(((.(((.(((((((((((...((((........
.(((((((......)))))))..))))..))))))))))).........((((
.(((((.(((((.((.(((.((.(((((((((......)))))))((((((..
.....((((((.(.......).)))))).(((((((....)))))))..((((
......))))))))))....)).)).))).))))).)).))))).))))))))
))..)).)))).)))..)))).)).))).......(.(((((((...((..((
(......))).))...))))))).)((.(((((.....)))))))....((((

((((........))))))))((((((.......))))))((((.(((......
)))))))).......((((.((...(((....)))...))...))))......
)).))))))))))))).(((((((........)))))))....(((((..((((
........)))).))))).))))))))).....)))))))))))))((((((((
....(((((((..((((....))))((((((((.....(((((...((..((.
...)).))..)))))....))))..)))).))).)))).....)).)).))))
...(((((((((....))))))))))))))).)))..))).

6.4. Designed Long RNA sequences

1. GGUUGGCAGGUGCGCCGCGGAAAGAAAACCGACGGCGUACCAACCGACCAAAC
AGGGUGACCGCACCGGCAACCACAGGCAAAAGCCAACCUCGGCGGGGAAAAAA
AAAAAAGAAAAAAGCGCACCGGACGCAAAAAGCGCCGGAGCGCCCCCGCCGAG
GGCGCAGUUGGAACCCGCGGGACAAACAAAAAACCCGUGGGCGGACAGCGCAA
AGUGGGCUGGACCCGGCCUCGGCAAAAGCGGGACCUAAAGCGGCCCGCCCGGC
AAACGCCGGAAAAAGUAUGGCAAGGGCCCAAACUGCCAAUAAGGCAGAGGGCC
CGGGGCUGCCGCCAGCAAGCCGCGCGGGCGAAAAAACGGCCCGUCACCACAAG
GGACAAAGGGCCGCGCCCAUACGCGGCCAGCCGGCAAGGUAGCACCCGCAGAC
AACCGCGAAAAGGGGCCAAACCGCAAAACGGCCCCAGCCUGGCUCCGACCUGA
AAAAAGGAAAAAGGGGCCAGGAAAACGCAUGGCGGAAAGGAGGCCGGAAGCGG
GGCCCCAGGUGGAAGUUUGCAAGGGCCCCAACGACAAGGCAAACAACAAGCCA
ACGGAGGGGCCCAAGCCAUGCACCCCGGAAAAAAAGACCGGGGAGCCGGGGCA
GAAGGGCAAAAGCCCAAGAAAAAAACCCGCCGGGGAGGACCGGCAGCGCCCAC
AAAAGAAGAAAAAAAAAAAAAAAACAAGGGCGCAAAAAAAAAGGCGGCCGGAAA
ACCGAGCAAAAGCCGGAACCGGCCGCAAAAAACAGUCAGGCCCGAGGCCCCAG
GCGCCCCGCACGGGCAAAGCCCGAAAGACGGGAGUGGGCCGGGGCCAUAGGAG
GUCCCAAAGCGCGGCGGAAACCCAAAAAAAAGGGAAAACCGCCAAAAGGGGCC
GCAAAAAAAGCGGUGUAACCUACAAAACCCGACGCGCGAAGGCGCGACGGGAAA
AGUAGGGCGCAGGGUCAACCGGCCACCGGCCGGCAACAAAAGCGCCAGAGCGC
GCGCCACAAGGGGCCAGGGCACUGAGAGGCCGGUCCCCCACGAGCGGGCGGGA
AACACCCGGAAGCAUCGUCGAGGAAAAAAAACCGACGCGUCCGUAAUCGAGUCG
CGCGGCAAAGAACCAGGGCGAUGCAACCGGGUGGCGGCACCCACAAAGUACCG
GUGGGAAGCCACCGGUGCAAAAAAAACAAAAAAA

2. AAUAAGGCUGCCCCCCGCAAAAAGCCGGGGCGUCGCGGGCAAGGAGGGCAGUA
AAGGGGUCGGAGCGGCCAAGCCUGCAACGCCCGGCCAAACGACUAGCAGAACA
AGCUGGACGAGGCCAAGGGCGAGGGAGGCAACCGAGCGGAAGGCAAAAGCUGA
CCGAAACUGACGGCACCGGCGGGCGCCAGGGGGAACCGAGCCAGGAUGCGCGA
GGCGCAGUGGGACAAAACCCGCCCGGCGGCAGAAAAAGCCGCUGGCGGAACGG
GACGGCCAAAAAAAGGCCGAACCGAACCGGCGAAGCGCCGGAAAAAAAAACCGG
CGCAAACGCAAGGAGCGGACCACCUCCGGCAGCUAGCUGGUGCGGCCCAGCGA
AACGACGGACUAAAUGCCGCGAACGCACGAGUGGAAAGACCACCGGGGCCGGA
GAAAGGGCUGGCAGCCAGGAGGAGGACCGCCGCAAUAAGCGCGCCCGUGGGCC
UGGCCGGCCCCCUGGAAAAAACAAACGCCCGCAAAAAGCCGCAGAACGGCAGC
AGCAAACCGCAGGGCGCCAACGAGUGCGAGUAGAGCCGCACCCCCAAAAGGGG
GAGCGGCCUGCUCUCACCGGGACGCGGCCAGCACCGACCCCGGGGACCGCGAA
AAGUGCGUUCAGAAGCCCAAAAACGACUGCCCGAAAAGCACCUCCCCCCAAGG
GCAAAAGCCCAAGAAAGGGCCACGGUAAACGAGGCCCAAGGGGGGUGGGCAAA
ACGAGGCAGGAGGAGGGCAAACUGGACGCGCAGGACGCGGUCACCCACAUUCG
GGGCACGCUAAAGGUCCCGAGUGCAGGCGAUGGUGUGAGCCCACCAGGCACCG
CGACGCCAAAGGCAGGCUGAAAAAAAGCCAAGCCAAGCCCGGGGGGCCCAAAA
AAGGCGGGCGGAACCGCCGAAAAAAAAAAAAAACACGAGCGGACCGAAUUAAAG
GGCAGCCCGCCCGCAAAAAAAGGCCGCAACCACGCGUUGGCGCAGGUCCGGGG
GGUGGGGCCAAGCCAAAGGCGGCCACCGCAGCCCCGGACCAGGGCCGACGAAA
ACGUGGACAAGCGGCCAGCACGGGCCCCGAGUGGAGGCUCCAAGGAGCCAAAG
GGGGCCGCAAAAAAAGACUAGUGGGAAAACGAUUAGGGGCCAGGCCAGGGGAU
CGGCAAAGCGCCAAGGGCGCAAGCUGAUCCGCAGGACGUGAGCACCGCGGCCC
CCGAGCAGCAAGGGCCCAAGCGAAAGCGGGCCCAAGCCGCAACCGCUCGGUGG
UCCGUGCAAGCCGGUGGGCGCCCACCCGCCAAGGGCCCCAAAAAACAAAAACC
GGGCGGUCGAGGAACGCUUGUGCACGCCAACCCGGCCCAAACAAGGAAGCCAG
GGGAGGCAUGCACGAGCGACCACGGCCAACCGGCGAAAAGUGGACCGGGGGA
AAACCCCCCGGAGGGGCCCAA

3. UAAAACCGAGUCCCACGCACCGCACGCGGUGACUGCCCGCCAAAAAACUGAGC
CGAGCAAGCUAGGCAAAUGCCUGGCGCUGAAUGCAAAAAAAAAGCGGUACGAA
AAAACCCCAGGACCCGAACAGCGCCGGAACACCGGCGGUGCAACGGGAAAACA
GGCGGUACCGACAAAGGCCCGCUUGAAAAAGCCUAAUGGCCCUGGGGAAACGU
ACCGCGCAGGGGGCGGAACCGUGUGGAAAAAAACCACACGGCCGAACCCCCUG
CAGAGGGUGCAGAAUAAGGAUCCUCGAAAGCGCUUCGGGAAAGCGGAGCGCAG
UGCUAAAGCACGCCGGCCAGAAAAAAGCCCGAAAACCGCCAAGAGGCGGAGGG
GCGGCGGGCAGGAGCCGCCCCAAAAAGGGGCGGCAGCGGUCAUCCCCCCCGGG
GAUGGCCGCAAAGUCUGGAAAAAAACCGUUCAAAGGCCAGAGGCCAAGAGCGG
CCAGGCACACCGUGUGCAAAAAAAGGAGCGAAAUAAGCGGCACGUCAGGACGC
GAACGGCCGCCCGCAAACCGGGGGCACAAAGCCCCCGGAAGCGGGUGGAACCG
GCGCGUCGGCCCUCGCAAAAAGGGCUGCGACAGGGCGGGCGGAGCGGACAGAC
GCAAAACCGCCUGCCCAGUCAAAAUAAGCAAACGCAGCAGCCCAAAAGCGCGG
GCCCUGAGGCGUCGCAAGCCCCCCGAGCAAAACCCCAAAAAGAAAGGGGAGGC
CGGGGGGCAAAAGCAGCGCCCUGGCCCAUGUCGGUUUCUCGAGGAGGAACCGA
CAUGGAAAGCAAAAGCAAAACCAGGGCAAGCGAAACAGGACCGCCGAACGGUG
GAACCUGCACGCAAAGCUUCGGCCACGGAAAACCGCCAGGAAAGCCAGGCGGA
AAAAGGCCGAAACUGGAGGGACCCGCGAGAGGCAAAGCGGUGUGAGCAAGCGA
CGCCCGACGGGCGACGCAGCACACACCGCAGCCAGUCGCAACCGGGGGGGCAA
CCGGGCGGCAAAAAAGCCGCUCAAAAAAGAGCAAGGCAGCUGGAAGAGCAGGG
CGAGGAGGGGCCAAGCCCCAAUGGGCGGCCCAAAACCUCGUCCAAGGAGCGGC
GAGAGGCCGCCCAAAGCGGCCCGGAGCUCCACCCGGGGCACUCUGGGAAAAAC
GAGAGGCCAAAGCCGGCCCAAACUCGGGGAAAAACUGGCGCAACCGCAAAAGC
GGGAGCAGCCAGAAAAGACCCCGAGGGGCCGGCAAACCGGUUGAAAAAAAACA
GCCGGGCGCGACCCAUCCGAGAGAGAUGAGGCGCGCAAGGGCCGUCCAGAAAA
AGGCCACCGUGGAAAAAAAAAAAAUCCGAAGCGCGGAGCUGAGGCGCGCUCCAU
GAAGUUGCGCAAAAGCGCAGCAACAUGGAGCGUGCCAGGGCACCGCAAGCUGC
AAAGAAAAGCCUGCGGCAAAAGCCGCAGGCGGGACCGGCACAC

4. UCAAAAAAGUGGGCCCACCGGGGCAAGCCCAAACCGCCUGCCCGCCAAAAAAA
GGCGGAAAAAAAAAAGCACCAAAAAAGGUGCAGGGGUGACGCGGGGCAAAACCG
CAAAAAACCCCGGCACCGCAGGCGAAAAACCGGACUAGCGUCCGGAAAAAAACG
CCAAAAGCGAACACGCAAGGCCCAAAAGGGAGCGGUGCAAGGGGCACCAAGCG
GGGCCGGACGCGCUUCCAAAAAACGGAAGCGCCCGAAGCCUCGCAAGGAGCCC
CAAAAAAAGCCAAGGCGGGAGGGGGACGCAAAAAGCGCCCCCACACCAAAAGG
UGAGGGAACAGCCCAAAUGGGUUGAAAGCGCUCAGGCGCAACUGGAAGGUGGG
GAGGCUCCCCACCACUCGGAAGCGGGAGGAGGGGAGGUCCCGCGGACAAGAAA
AAAAAAAAAACCGCGGGACACCGCAAAAGCGAAGAUGGGCGGAGCAAACGCCC
GAAACCCAUCACCUCAGCCACCACCGGAAAAAAAACCCGGCAUAGCCGGGAAA
ACCCGCCAAGGCAAGGCGGACGCAAAAACCGGGGAAACACCCCGGGAAGCGUU
CGCCAGACACGCAAAGCGGUCAGCCCAGACCUCAGCGGAGUAGCCGCCAAAAC
CGGCAAAAAGCCGGACGGCGGCUAGAAAACCAAAAGCUAAUGCAGCUGAGGAA
GGGCAAAGGGCAGCCAUGAGACAAAACCGGCGAGAGGAAAGCGGACAAAAAAA
CCGCCCAGUCGCCGGCAAAGGGCAGGGACGGACGGCGCCGGGUGAAAUAAAAC
ACCCGGCGCCCCGACCCGAAGUCCAAAAAGCGCCGGCCAAGCCGCGCCGGGCA
AAAAAAAAAACCACAAAAAGUGGCGCCGCCAGGCAAAAGCCGCUCCGGACUCU
GGAAAAGCCAAGCCUAAGGCAAGGCAAAGCCGGCGCCGGUGCUAAAACCGGAG
UUCCGCAGAAGCGGAGCACGGCGGCAGCAAAAAGCCCCGCGCCGGCGCUGCCG
CCACAAGGCGAGCGGCAAAAAACCGAGCCGCGAAAAGGCCCCGGGUUCGCUAC
GUCCGCGGACACCGGGAAAAAAGCCAAGACCGCGGAAAGGAAGCCGGCUCGGG
GAAAGCAGCCGGGAGCGACGCGGGGCCUAUGGCACCCGGGCCUCCAAGGCGGG
GGCCCAAAGGCGCGGACGAGGCCGGCGCAAAGAAACAGCCAAAAGGCUGCCGC
CCCACAAGGGAAAAAGCGGGUCCAUGGCGCCCAAGGCCCGGAAAGAAAACCGG
GCCAAAACGGGGCAAGCGAAAAAAGCCCCACGGAGCCCCAGCCGGUGGAGGGC
GCGCCGCCCGGACCGGAUGGAGCGGGCAAAAAAAAAAGGUGCAGCCCUGGAGG
GCCGGCAAAUAGCGCCAAACGGCGCGCCCCCGAGCCGCAAAAGCGGCGCGGAG
GGAGAGAAACCCUCCGCAAAUAGUACGGCCGCAGAAGCGGUCGUACACAAAGG
GGGCAA

5. CCUCCGGCCGGCCAGGACAAACGGCGGGCCAAGAGCGAGGGACCGACCGGAGC
CGGAAAAAGCAGGGGAGGGGUCGAGCGACCCCUCUCCAGCAGCGACAUCGGAC
GGAACCAGGCUCGGCCGAGCGCCGGCGGCGGGUGGAACCGGGGAAAACCCCGG
AAACCGCUCGCCACCGCGCCGGAGGAAAAAACCCCGGCGCGGAAAACAGCCCU
GCUGGCCGGCUCUCGGACCAAGCGGAAGAGCGAACCGGCCGCAAAACAAGCGG
CUGGCGCAAACUUCUGCAGGCGGCGCAAAAGCGAAACCGCCAGAAGGCCGACA
AACAAAAGUCGGCCACCUCAUAAGAGGGGCCCAGCCCCGAAUAAAACGGAAAC
AGCCAAAGCUGAACCUGGUCGGCCGGAGGAACCACCGCUGCAAGGGGGGGCAC
CGCCUGAGAAAAACAGGCGGGCCCCAACACCGCGAAAACCUGCACUUGAAAUC
AAGGCAGCAAACGUGGGUGGCCCGGCCAAAGGCCGGAAAGAGCCACAAAAACA
CGCCGGGCUCCCUUGUGGACCGGUUACGGGCCGCGUUAUGAAUAACAUAACGC
ACGUGUAGCUGGAACCGCGAGGAAACCGCUGCGAAAGGGCGGCUCCUGCCGCC
AAACCUGCGCAAAACCAGGAAGACGCCUGGAAGCGACAGGGGCAGGCCGGUGC
CGCCCAAAGGGGGCGGAAGAGCGUUGCGUGAAGGCGCCGCAGCGAAAGCGGAA
AAGAAAACCGCCGAAGCGGCGCCAAAAAGCGGCGGGGAAAGUCUUCGUCUGCG
GCCAUGCCGCAGACGAAGGCGCCCAGAAAGCGCCACACCCCGGGCGCAAACCG
GAGUUGGUAAUCCAGCAACCGGAAGCGAAACCCGGGGCAGGCAAAAAAGUCGC
CGGCCCUGAAGGAGCAGGCCCAAAAAAACGGGCAGCUCAAAGCGGUCGCCGGG
CGCCGUGGGCGCGGAAAGAAAACCGCGCAGCCCCCACGGUGCACGGCCGCAAC
CAGCGGCCAGCGCCGAGGAGGGAAAAACCGCGGCGCAACCGAGCCGCCCAGUC
AAAAAGACAGGCGGGCGGGCCAACCGACCGCUAACGCGAGUCGGGGCCACGCC
CGCCGGGCUGGCAAGCGAAGCCAAAAAAAGGCAGACGCAAGCCAGACCCGGGA
CGGCAAAAUGCGACCGCAACAAACUUCAGGGAGAAAGCGCGUGAAAAGCCCGC
GGCGGUGCUAAAGCACCACCGCAAGGGAAAAGCACGCGCAAAGGUCCGAGCCC
GGGGAAAACAGACCCCGGGAAAAGGUCCAAAAGGGGAAACCGCGCGGAAAUAA
ACCGCGCGGAAACCCCAAACCAGUAAAUCUGAAAACCGGCGACAAGUGGAGGG
AGGCCGCGCAAGGUGACGGGCGGGUCCCAAGGACCUGCCCAACACCAAGCGCG
GCAACCCGCCACGAAAAAAGAAGAAAACAGGCCGCCAAAAGGCGGCCUGGCAA
AGUGGGCA
